# Emerging advances in drug delivery systems (DDSs) for optimizing cancer complications

**DOI:** 10.1016/j.mtbio.2024.101375

**Published:** 2024-12-05

**Authors:** Kerui Li, Bei Guo, Junmou Gu, Na Ta, Jia Gu, Hao Yu, Mengchi Sun, Tao Han

**Affiliations:** a Department of Medical Oncology, The First Hospital of China Medical University, Shenyang, 110001, China; b Department of Endocrinology, General Hospital of Northern Theater Command, Shenyang, 110001, China; c Department of Hepatobiliary and Pancreatic Surgery, The First Affiliated Hospital of Zhengzhou University, Zhengzhou, 450000, China; d Department of Neurology, Second Affiliated Hospital of Dalian Medical University, Dalian, 116044, China; e Department of Otolaryngology, The First Affiliated Hospital of China Medical University, Shenyang, 110001, China; f School of Pharmacy, Shenyang Pharmaceutical University, Shenyang, Liaoning, China

**Keywords:** Drug delivery systems (DDSs), Tumor complications, Inherent complexity, Quality of life, Survival rates

## Abstract

The management and treatment of tumor complications pose continuous challenges due to the inherent complexity. However, the advent of drug delivery systems (DDSs) brings promising opportunities to address the tumor complications using innovative technological approaches. This review focuses on common oncological complications, including cancer thrombosis, malignant serous effusion, tumor-associated infections, cancer pain, and treatment-related complications. Emphasis was placed on the application and potential of DDSs in mitigating and treating these tumor complications, and we delved into the underlying mechanisms of common cancer-associated complications, discussed the limitations of conventional treatments, and outlined the current status and potential development of DDSs for various complications in this review. Moreover, we have discussed the existing challenges in DDSs research, underscoring the need for addressing issues related to biocompatibility and targeting of DDSs, optimizing drug delivery routes, and enhancing delivery efficiency and precision. In conclusion, DDSs offer promising avenues for treating cancer complications, offering the potential for the development of more effective and safer drug delivery strategies, thereby improving the quality of life and survival rates of cancer patients.

## Highlights

• Management of cancer complications is as important as treatment of the primary lesion in oncological care.
• Innovations in Drug Delivery Systems (DDSs) for Cancer Treatment Improve Efficacy and Reduce Side Effects.
• Applications and challenges of DDSs in the treatment of different cancer complications.

## Graphical abstract

This abstract graphic is coordinated with the abstract section illustrating the application of drug delivery systems in different tumour-related complications.

**Figure:**
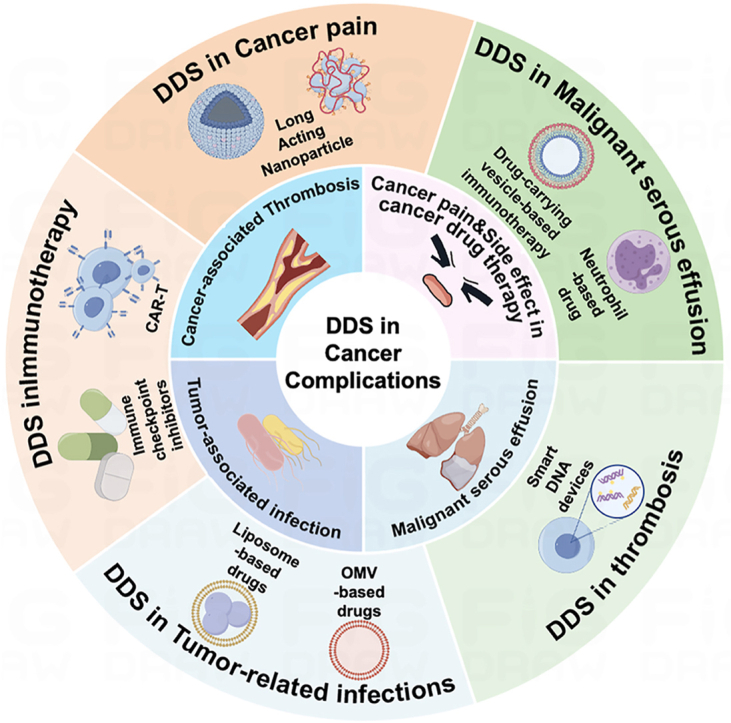

## 1 Introduction

Cancer is a chronic disease that seriously affects the quality of life of patients and poses a major health challenge and economic burden worldwide [1]. In addition to combating aggressive cancer cells, doctors and patients must confront a range of challenging complications. These complications may stem from the natural progression of the disease or arise during the course of treatment. In some cases, cancer complications may even be the initial symptoms experienced by patients [2]. Disturbingly, statistics reveal that approximately 4 million cancer patients seek treatment in emergency departments annually due to various tumor-related complications [3]. Older patients, in particular, face an increased risk of postoperative complications, intolerance to radiotherapy, and disease progression due to the complex nature of their cancer and treatment [4]. Cancer complications, these covert adversaries, not only significantly reduce patients’ quality of life and complicate treatment but also decrease survival time and pose life-threatening risks. Consequently, prevention and timely management of these complications plays a vital role in conquering tumors [5].

At present, the commonly used clinical treatments for cancer complications are mainly conservative pharmacological treatments [3], interventional treatments and surgical treatments. However, interventional treatments, surgical treatments, and these traditional treatments often cause different degrees of therapeutic side effects to patients. For instance, thrombolytic drugs for blood clots can easily lead to high-risk bleeding, unstable blood pressure and other therapeutic side effects [6]. Similarly, opioids or non-steroidal anti-inflammatory drugs (NSAIDs) commonly used in the treatment of cancer pain can result in gastrointestinal reactions and in severe cases, cause psychoneurotic symptoms [7]. Moreover, chemotherapeutic drugs may damage the haematopoietic system, resulting in symptoms such as bone marrow suppression and anaemia, or gastrointestinal symptoms such as loss of appetite, vomiting and diarrhoea [8], or even neurotoxicity of the central or peripheral, among a variety of other adverse reactions. Last but not least, targeted drug therapies can carry the risk of cardiotoxicity and skin toxicity [9], while patients receiving anti-angiogenic therapy may face hypertension, haemorrhagic stroke, and unstable angina [10]. Hence, to improve patients’ quality of life and extend clinical survival, it is imperative to explore more effective treatment modalities that minimize the incidence of treatment side effects.

Research has illustrated the targeting benefits of nanoparticles in drug delivery compared to conventional drugs, thus enhancing drug utilization efficiency and efficacy, and mitigating toxic side effects [11]. The rapid development in the field of pharmacotherapy is attributed to the continuous innovation in drug delivery technologies. As therapeutic approaches have evolved from traditional small molecules to proteins, peptides, monoclonal antibodies, nucleic acids, and even living cells, drug delivery technologies have also advanced to meet new challenges. Drug delivery systems (DDSs), represented by nanoparticles, can precisely control the distribution of therapeutic agents within the body in terms of dosage, timing, and spatial dimensions, ensuring that the drug is delivered to the specific site at the right time. As an emerging field of technology, DDSs have shown significant potential for application in the treatment of oncological complications [12]. Through the implementation of targeted delivery and controlled release technologies, DDSs can significantly improve therapeutic efficacy, mitigate toxic side effects and provide new opportunities for individualised therapeutic strategies [13].For instance, pH-responsive systems leverage the pH changes in the gastrointestinal tract to achieve targeted release, enhancing therapeutic efficacy while reducing damage to off-target organs. DNA nanomaterials have addressed the issues of premature leakage of chemotherapeutic agents during delivery or failure to release upon reaching tumor cells, significantly reducing the potential biological toxicity of drugs and accomplishing efficient drug delivery. The progress and refinement of drug delivery technologies have spurred the clinical adoption of numerous novel drugs, with associated preclinical investigations and clinical applications advancing steadily. These innovative and improved technologies are poised to amplify the effectiveness of drug therapy and enhance the overall patient therapeutic experience [14]. This review aims to describe and summarise the mechanisms of various complications, analyse the treatment modalities of different complications, and explore the application and development prospects of drug delivery.

## 2 Tumor-related complications

Currently, clinical research focuses more on tumor control and primary tumor treatment, with less attention given to complications during tumor progression and anti-tumor treatment.Given that some complications cannot be cured, the focus may shift to symptomatic treatment [15]. It is distressing that these complications will not only lead to certain damage to the patient's normal tissues or cells during anti-tumor therapy, resulting in various adverse reactions, but also cause great physiological and psychological burdens on the patient, reducing their quality of life and treatment adherence, and complicating their overall condition [16].

### 2.1 Silent killer--cancer-associated thrombosis (CAT)

Complications associated with CAT, encompassing arterial or venous thromboembolism and disseminated intravascular coagulation, typically occur in patients with clinically active malignant tumors. CAT persist throughout the oncological diagnostic and therapeutic journey in these patients and represents a primary cause of mortality, surpassing the impact of cancer itself, with a prevalence nine times higher than in non-oncological patients [17]. The mortality rate for cancer patients with venous thromboembolism (VTE) is 2–3 times greater than those without VTE. Furthermore, anticoagulant therapy for VTE events in patients with solid tumors is complicated due to the elevated risk of both thrombotic recurrence and bleeding during anticoagulation treatment [18].

#### 2.1.1 Mechanisms of formation of cancer-associated thrombosis

**Fig. 1:**
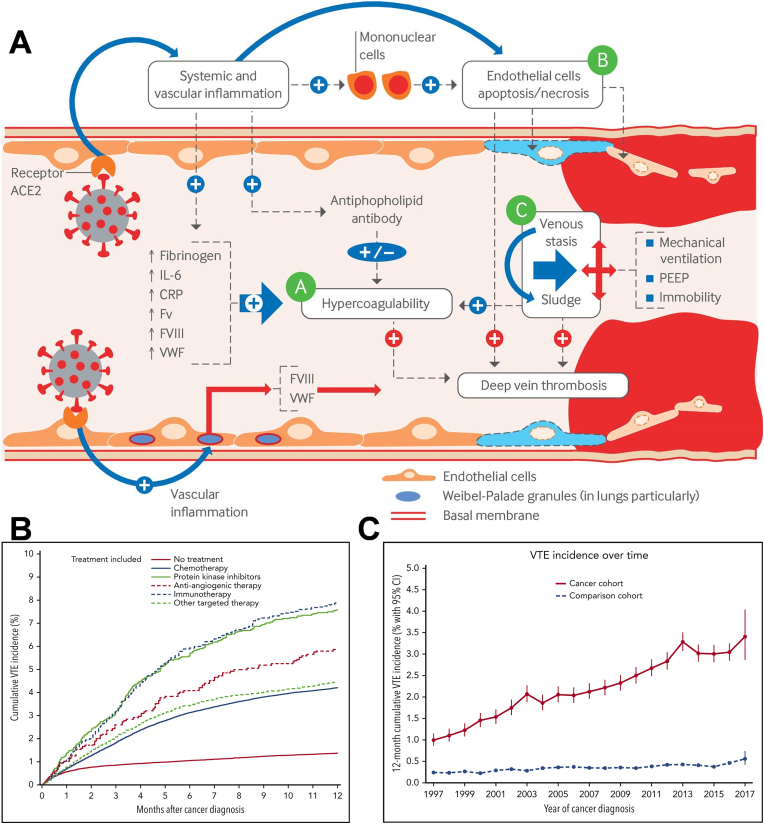
**A.** Pathogenesis of venous thromboembolism [203]. Copyright 2023, BMJ **B.** Twelve-month cumulative incidence of VTE in cancer patients receiving systemic therapy during the first 4 months after cancer diagnosis. **C.** Twelve-month cumulative incidence of VTE in the cancer and comparison cohorts between 1997 and 2017. Reproduced with permission [204]. Copyright 2021, Elsevier.

In 1856, Rudolf Virchow introduced a triad believed to contribute to thrombosis, which includes endothelial damage, stagnant blood flow, and a hypercoagulable condition [19]. While the mechanisms and risk factors for thrombosis in malignant disease states are likely similar to those in non-malignant disease states, the hypercoagulable state in malignancy may be driven by cancer-specific pathways. The presence of malignancy typically increases the coagulation cascade response and platelet activation, as well as circulating levels of certain blood cells (e.g., platelets and leukocytes) [20]. Additionally, anti-tumor therapies can further heighten the risk of thrombosis through direct or indirect pathways [21]. These treatment-related factors include hospitalisation, radiotherapy, surgery, anticancer drugs, chemotherapy, and targeted drugs (Fig. 1A) [17,22]. Moreover, the frequency of CAT varies depending on the primary tumor site, with several studies indicating an elevated VTE risk among patients with pancreatic [23], lung [24], and brain cancers [25]. The cancer stage is also significantly correlated with an increased risk of CAT, with individuals suffering from metastatic disease exhibiting the highest susceptibility to VTE development (Fig. 1B–C) [17,26]. Furthermore, patient characteristics such as age, gender, past medical history, and the presence of other comorbidities are also influential factors that contribute to hypercoagulable states in cancer patients [27].

Tissue factor (TF) stands as the most extensively studied procoagulant protein originating from tumors, with its expression levels often mirroring the aggressiveness of tumors [28]. Tumor-derived TF plays a central role in fostering prothrombin production within cancerous tissues, while concurrently fostering tumor advancement through direct modulation of vascular endothelial growth factor expression in both malignant and host vascular cells [29]. TF expression has been identified in a variety of solid and hematological tumors, including pancreatic, brain, colorectal, lung, breast, ovarian cancers, and myeloproliferative neoplasms (MPNs) [30]. Microparticles (MP) released by tumor cells can act as different contributors to the mechanism of cancer thrombosis. Studies across various cancer types have demonstrated that circulating MPs expedite thrombus formation in vivo [31]. Tumor cells are known to express TF and release TF-positive MPs into the circulation. Numerous studies have found a correlation between increased levels of TF-positive MPs and venous thrombosis in individuals with cancer, suggesting their potential as a biomarker for identifying high-risk patients [30c]. Cancer-associated fibroblasts express podoplanin protein, which triggers platelet activation and aggregation via C-type lectin receptor 2 (CLEC-2) [20,32]. CLEC-2 expression is also observed in tumor cells, inflammatory macrophages, and cancer-associated fibroblasts, thereby fostering cancer progression and metastasis [33]. Podoplanin proteins, released by cancer cells into the bloodstream, exert influence thrombosis at remote sites [34]. Additionally, a study linked podoplanin expression in gliomas with altered indicators of coagulation activation and heightened the risk of venous thromboembolism [35]. Moreover, tumor-derived microparticles carrying podoplanin proteins have been detected in the blood and circulation of patients with pancreatic and colorectal cancer [36]. Pancreatic cancer cells exhibit high expression levels of plasminogen activator inhibitor 1 (PAI-1), a key negative regulator of the fibrinolytic system [37]. Elevated levels of PAI-1 are known to be associated with thrombosis. However, few studies have assessed plasma PAI-1 in cancer patients. Two studies have reported higher plasma PAI-1 levels in patients with pancreatic cancer and high-grade gliomas compared to healthy controls [38]. Another study showed that increased levels of active PAI-1 independently correlated with a heightened risk of VTE in patients with pancreatic cancer [39]. Cancer procoagulant (CP), a cysteine protease first discovered in rabbit tumors, initiates coagulation by directly activating factor X without depending on factor VII [40]. A study on breast cancer patients reported that CP is unrelated to procoagulant markers [41]. The extent of CP's role as a procoagulant in cancer remains largely unexplored, necessitating further research to validate its function in coagulation activation and its association with cancer-related thrombosis. Adenosine diphosphate (ADP) and thrombin are well-established platelet aggregation agonists. Cancer cells secrete ADP, activating platelets and causing aggregation via P2Y1 and P2Y12 receptors [42]. Elevated levels of thrombin, detected in the plasma of pancreatic cancer patients, suggested the involvement of tumor-derived products in platelet activation and coagulation within pancreatic cancer [43].

#### 2.1.2 Treatment of cancer-associated thrombosis

Anticoagulation continues to play a crucial role in the therapy of venous thromboembolism in cancer patients. In the selection of appropriate medication for patients, factors such as tumor type, bleeding risk, gastrointestinal tolerance, and drug interactions need to be thoroughly considered. Low molecular weight heparin (LMWH) has demonstrated superiority over warfarin in reducing VTE risk in cancer patients and is now the recommended standard thromboprophylaxis. The CLOT trial, a randomized controlled study, compared LMWH monotherapy to VKA in preventing and treating VTE in active cancer patients. Dalteparin demonstrated superior efficacy compared to VKA, reducing symptomatic recurrent VTE without increasing major bleeding risks. Thus, it is recommended as the initial therapy for cancer-related thrombosis according to numerous influential clinical guidelines [44]. However, the inconvenience of injections remains a major barrier to LMWH therapy in clinical care for many patients. Direct oral anticoagulant (DOAC) therapy is currently approved for venous thrombosis, and a randomized clinical trial suggesting comparable efficacy and safety between DOACs and LMWH in cancer patients, although indirect comparisons were relied upon [45]. An inaugural large randomized prospective clinical trial compared the safety and efficacy of dalteparin (a low molecular weight heparin) with edoxaban, a direct-acting factor Xa inhibitor [46]. Oral edoxaban demonstrated non-inferiority to subcutaneous dalteparin in preventing recurrent venous thromboembolism or major bleeding. Although the edoxaban group showed a higher incidence of major bleeding, it also experienced a lower rate of recurrent venous thromboembolism. Compared to LMWH, vitamin K antagonists (VKA) are less effective in preventing cancer-related recurrent venous thromboembolism [47]. VKAs pose several constraints, including necessitating frequent laboratory monitoring and dosage adjustments, interacting with numerous medications and dietary elements, and exhibiting a slow onset and offset of action. Therefore, VKA is not the recommended therapy for CAT according to leading guidelines. Despite this recommendation, VKA remains commonly used in CAT patients due to its cost-effectiveness, oral administration, and widespread familiarity among healthcare providers. Prolonged use of VKAs like warfarin may lead to osteoporosis. Despite the common occurrence of venous thromboembolism in oncology patients and its contributes to mortality, there is still limited research on meeting the unique needs of patients with respect to anticoagulation therapy. Optimal dosage, duration of therapy, and drug interactions are still poorly understood, highlighting the urgent need for more effective and safer antithrombotic therapy.

Anti-thrombotic drugs play a crucial role in the prevention and treatment of thrombosis-related diseases, but they also have some limitations, such as a narrow therapeutic window, drug interactions, bleeding risks, individual response variability, compliance issues, and the monitoring and adjustment of anticoagulants. These limitations may lead to poor treatment outcomes or increased bleeding risks for patients, affecting their quality of life and prognosis. Therefore, future research directions may focus on developing safer, more effective, and convenient anti-thrombotic drugs, as well as personalized treatment strategies based on genotype and other biomarkers to improve therapeutic effects and reduce side effects. At the same time, the development of new anti-thrombotic drugs and treatment methods, such as direct oral anticoagulants (DOACs) and gene therapy, is also expected to provide more effective treatment options. In summary, the limitations of anti-thrombotic drugs have prompted researchers to continuously explore new treatment methods in the hope of providing safer and more effective treatment plans for patients.

### 2.2 Malignant serous effusion

**Fig. 2:**
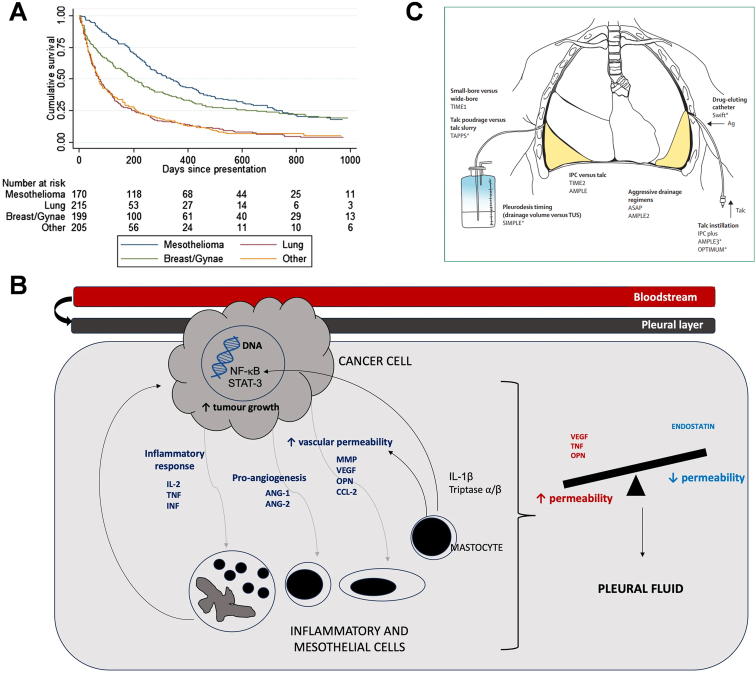
**A.** Kaplan–Meier survival curves according to cell type for the UK, Australian and Dutch cohorts combined [205]. Copyright 2014, BMJ **B**. MPE pathogenetic mechanism [206]. Copyright 2024, Respiratory Research. **C.** Recruited and recruiting trials in management of malignant pleural effusion Ag = silver. TUS = thoracic ultrasound. *Recruiting trials [207]. Copyright 2020, Elsevier.

Serous effusion is the accumulation of fluid in the plasma lumen of the pleura, peritoneum, and pericardium. The imbalance between the amount of fluid produced and absorbed in tumor patients under pathological conditions leads to patients developing malignant pleural effusion (MPE) or malignant ascites (MA). Most solid tumors produce pleural effusions peritoneal effusion or even pericardial effusion at later stages of progression, e.g., gastric, breast, lung, and liver cancers. The presence of MPE or MA is an ominous prognostic sign for cancer patients because the presence of MPE/MA indicates a tumor that is not curable by surgery and has a short life expectancy. The occurrence of MPE/MA can result in adverse effects like dyspnea, persistent pain, abdominal distention, peritoneal inflammation, and intestinal obstruction, significantly affecting the patient's quality of life. Approximately one out of every six patients with malignant tumors will develop pleural effusion during the course of their illness, with a median survival time of less than 12 months (Fig. 2A) [48]. Despite these observations, the molecular mechanisms underlying effusion generation remain unclear.

#### 2.2.1 Mechanisms of formation of malignant serous effusion

Vascular endothelial growth factor (VEGF) is now recognised as a crucial regulator of tumor angiogenesis, and plays a significant role in the entire process of tumor growth by stimulating tumor angiogenesis, activating host vascular endothelial cells, and promoting malignant proliferation. VEGF has attracted interest as one of the important mediators found in malignant effusions due to its central role in pleural effusions [49] and its potential as a therapeutic target [50]. It was observed that various cancer types exhibit increased expression of VEGF, a phenomenon linked with poor prognosis in pancreatic cancer [51], gastric cancer, colon cancer [52], lung cancer [53], breast cancer [54], prostate cancer [55] and melanoma [56]. Factors regulating VEGF expression encompass hypoxia, multiple growth factors (e.g., epidermal growth factor, transforming growth factor, insulin-like growth factor, etc.), various hormones, as well as the activation and dysfunction of proto-oncogenes and tumor suppressor genes driven by oncogenic mechanisms [57]. To elucidate the involvement of TNF-α in the formation of MPE, Georgios T. Stathopoulos and colleagues investigated its role in preclinical models and its impact on pleural tumors through in vitro experiments. The study revealed that TNF-α induced the production of TNF-α and VEGF through nuclear factor κ b and neutral sphingomyelinase-dependent pathways, respectively, thereby in turn promoting angiogenesis. Moreover, TNF-α promoted the action of VEGF by enhancing vascular permeability. The findings also revealed that TNF-α directly promotes the survival of tumor cells in vivo by limiting apoptosis, contributing to the formation of MPE [58]. These results underscore the intricate role of TNF-α in the pathogenesis of MPE, suggesting its potential as a therapeutic target for interventions aimed at addressing this pathological condition (Fig. 2B) [59].

#### 2.2.2 Treatment of malignant serous effusion

**Fig. 3:**
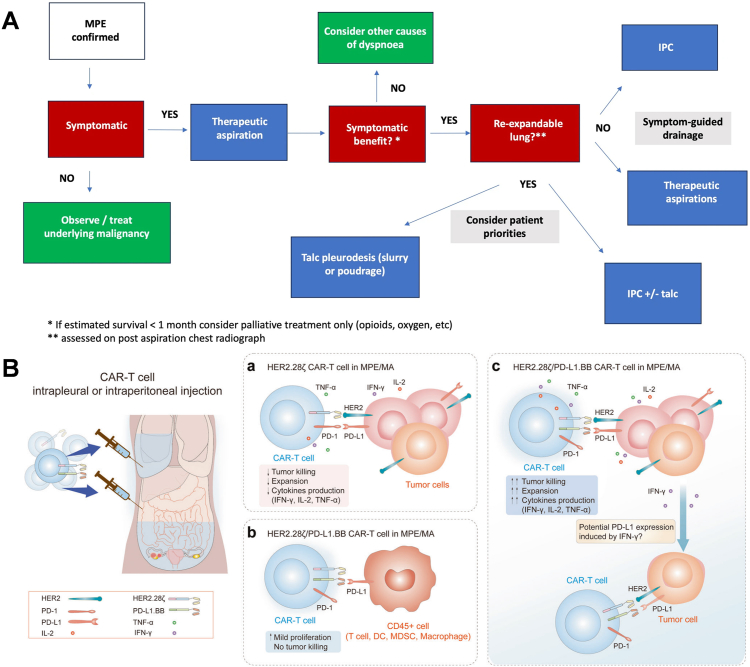
**A.** Management approach in MPE**.** [206] Copyright 2024, Respiratory Research**. B.** A virtual treatment scenario of CAR-T cells in pleural/peritoneal cavity. a–c. The therapeutic pattern and potential mechanism of regional delivery of HER2.28ζ a and HER2.28ζ/PD-L1.BB CAR-T cells b, c to treat pleural or peritoneal metastasis accompanied by MPE/MA. Reproduced with permission [208]. Copyright 2022, Springernature

The primary objective of MPE treatment is to shrink the tumor size and drain pleural fluid to relieve symptoms such as dyspnoea, cough, and reduce the number of days of hospitalisation and improve the patient's quality of life. A wealth of research data over the past decade have demonstrated the efficacy of the indwelling pleural catheter (IPC) as a first-line treatment for the management of recalcitrant MPE. Although IPC was traditionally used as a second-line treatment after failure of pleurodesis or sclerotherapy, new clinical trial results support its use as an effective preferred method of controlling dyspnoea [48b]. The TIME2 trial [60] and the AMPLE trial [61] compared the efficacy of IPCs versus talc pleurodesis in alleviating dyspnea and shortening hospital stays. The findings indicated that there was no significant disparity in relieving patient-reported dyspnea between IPCs and talc pleurodesis among patients without prior thoracic interventions. However, IPCs did reduce hospitalisation duration, albeit with a relatively higher incidence of adverse events in the IPC group. The ASAP trail [62] and the AMPLE-2 trail [63] further compared different IPC drainage regimens and found that an aggressive daily drainage strategy increased the incidence of self-reported chest pain and earlier catheter removal but did not differ significantly from symptom-guided drainage regimens in relieving dyspnea. In addition, IPC Plus [64] found that combining talcum powder treatment with IPC administration was superior to IPC treatment alone among patients without lung entrapment. Several trials in recent years are summarized in Fig. 2C below. Although these findings contribute to refining the management of MPE (Fig. 3A), shortening hospital stays, and enhancing patient quality of life, these approaches are associated with a notable incidence of adverse events [59]. The application of diuretics and puncture and drainage can only alleviate the patient's symptoms, and the local puncture to draw fluid or drainage and intracavitary administration of drugs are the main means of treating malignant pleural and abdominal cavity effusion in the clinic, but they can not reduce or eliminate the formation of effusion fundamentally. Moreover, frequent punctures and the release of large amounts of fluid can often lead to complications such as electrolyte disorders, local infections, pain, plasma membrane thickening, and encapsulated fluid, leading to hypoalbuminemia and wasting. In recent years, there has been exploration into the local administration of cytotoxic and immunological drugs to enhance treatment outcomes for effusion. Professor Wang's team has developed a novel PD-L1-targeted chimeric switch receptor (PD-L1.BB CSR) aimed at augmenting CAR-T cells' ability to identify and eliminate tumor cells, thereby enhancing therapeutic efficacy (Fig. 3B) [65]. This suggests that relying solely on palliative care is insufficient in preventing effusion formation, highlighting the imperative for an ultimate therapeutic goal centered on active tumor control. Although current clinical treatments for pleural effusion are limited and the therapeutic outcomes remain unsatisfactory, and the search for new therapeutic modalities with low rates of adverse effects and high rates of efficacy should not be overlooked.

### 2.3 Tumor-associated infection

Studies have indeed demonstrated that infection is one of the main causes of morbidity and mortality in patients with malignant tumors. For cancer patients, infection is actually more frightening than metastasis. When an infection occurs, it not only adds to the patient's suffering but also impacts the effectiveness of therapy and prognosis, and serious infections can even be life-threatening. Furthermore, there are instances where the malignant tumor itself may predispose the patient to serious or recurring infections [66].

#### 2.3.1 Mechanisms of formation of tumor-associated infection

**Fig. 4:**
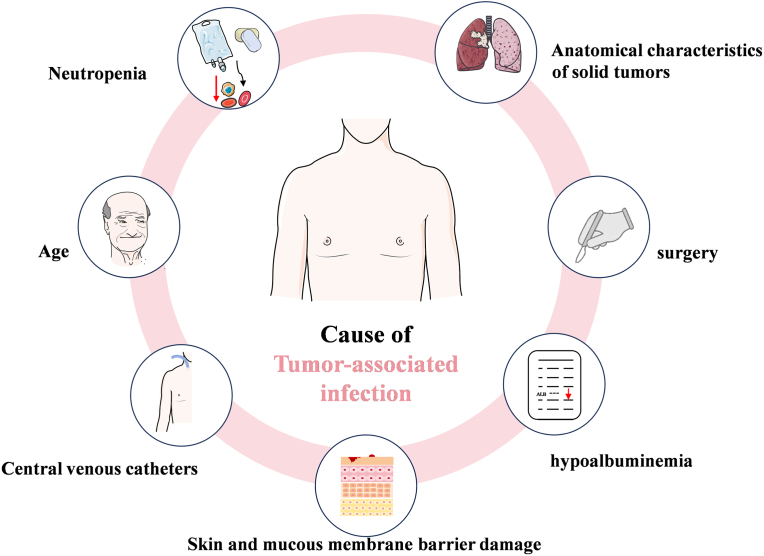
Cause of tumor-associated infection.

Neutropenia has long been acknowledged as a significant risk factor for infection development in patients undergoing chemotherapy. When the neutrophil count drops below 500/mcL, defining neutropenia, susceptibility to infection increases. The incidence and severity of infections exhibit an inverse correlation with the neutrophil count, with the highest risk of severe and bloodstream infections observed when the neutrophil count fell below 100/mcL [67]. While neutropenia remains a pivotal risk factor for infections, other conditions that compromise the immune system also contribute to infection susceptibility in oncology patients. These conditions include mucosal barrier damage, splenectomy or functional asplenia, prolonged use of central venous catheters (CVCs), and structural alterations in solid tumors, each contributing to varying degrees of immune suppression and thereby augmenting infection incidence. Specifically, maintaining the integrity of the mucosal lining in the gastrointestinal, nasal, and genitourinary tracts is crucial as it serves as the primary barrier against invasion by a diverse array of pathogens. However, chemotherapy and radiotherapy may damage these mucous membranes to varying degrees, reducing their immune function and making it easier for local flora to invade the body and cause infection [68]. As early as 2014, a retrospective analysis found that long-term use of CVCs has been associated with increased risk of infections in elderly cancer patients, and that CVC exposure could triple the risk of infection in pancreatic cancer patients and even increase it sixfold in breast cancer patients [69]. The anatomical characteristics of solid tumors may also predispose patients to infection. Tumors in areas with a rich blood supply may overgrow and cause local necrosis and infection foci. Bronchopulmonary carcinoma may cause recurrent obstructive pneumonia due to obstruction of the airways, while abdominal tumors may obstruct the genitourinary tract or the hepatic and biliary ducts, leading to pyelonephritis or cholangitis and gallbladder inflammation. Additionally, patients undergoing surgery for malignant tumors may face an elevated risk of infectious complications attributed to various factors including the nature of the surgery (such as high-risk procedures like oesophagectomy and hepatobiliary reconstruction), tumor burden, preoperative health status, and history of prior surgery, chemotherapy, and radiotherapy [70]. Combined with weakened organ function and nutritional exploitation by the tumor, hypoalbuminemia can cause immune decline, reducing the effectiveness of treatment and increasing mortality rates [71]. Older cancer patients, who are more physically fragile and experience a pronounced decline in immunity, are especially susceptible to bacterial infections, further increasing the probability of infection (Fig. 4).

#### 2.3.2 Treatment of tumor-associated infection

The use of antibiotics is currently central in the treatment of infections, involving empirical therapy and targeted therapy. Targeted therapy usually adopts de-escalation therapy, whereby the use of drugs is adjusted in a timely manner according to biomedical indicators in order to accurately control the duration of treatment and the time of discontinuation; whereas empirical therapy relies on the clinical experience and judgement of the doctor [72]. The conventional primary routes of drug administration persist as oral or intravenous. Nevertheless, various drug delivery modes offer distinct advantages and limitations in disease treatment. Oral medications boast high patient compliance and ease of use, yet they necessitate absorption through the gastrointestinal mucosa, presenting challenges in mucosal penetration and drug stability, particularly for poorly soluble drugs [73]. Intravenous administration, a systemic route of drug delivery that bypasses the gastrointestinal barrier, necessitates high doses to attain the desired therapeutic effect. However, antibiotics can impact the function of normal organs, such as the heart, liver, or kidneys, and lead to issues like phlebitis and bacterial resistance with prolonged use. Therefore, individualised risk assessment, prophylactic measures, as well as the timely recognition and treatment of active infections are important components in cancer management to optimize treatment outcomes [74]. In clinical practice, physicians need to consider the choice of treatment regimen, the mode of drug delivery, and the patient's specific situation to ensure optimal therapeutic efficacy and minimal side effects.

The routine treatment for cancer-related infections primarily relies on antibiotics, but there are some limitations. Firstly, the overuse or misuse of antibiotics can lead to increased resistance, making infections harder to treat. Secondly, antibiotic therapy may disrupt the balance of normal flora, leading to secondary infections such as candidiasis. Moreover, for patients with neutropenia, controlling infections is challenging even with antibiotics, as their immune systems are severely compromised and struggle to effectively combat infections. The long-term use of intravenous catheters, while convenient for treatment, also increases the risk of infection, especially for elderly patients or those with underlying diseases. Therefore, there is a clinical need for more precise treatment plans, as well as meticulous infection risk assessments and preventive measures for patients, to reduce the occurrence of cancer-related infections and enhance treatment outcomes.

### 2.4 Cancer pain

Cancer pain is a cancer-related symptom that can occur at any stage of cancer and is not limited to advanced stages. Despite notable advancements in pain management, effectively managing cancer-related pain remains a formidable challenge. Inadequately controlled cancer pain not only profoundly impacts the quality of life for oncology patients but also disrupts the progression and effectiveness of antitumor therapies [75]. A systematic review analyzed published literature on cancer pain management from 1994 to 2013, and by updating the 2008 systematic evaluation [76], the researchers found that compared to the pre-2007 period, based on a comparison of pain management index (PMI), the under-treatment rates among cancer patients declined after 2007; however, approximately one-third of patients still did not receive adequate pain medication in proportion to their pain intensity [77]. The causes of cancer pain are complex and diverse, and can be broadly classified into the following three categories: 1) Tumor-related pain, which may result from direct invasion and compression of local tissues by the tumor or metastasis of the tumor to bone or other soft tissue nerves. 2) Pain caused by anti-tumor treatment, such as surgery, invasive treatment, radiotherapy, etc. 3) Pain caused by non-tumor factors, including comorbidities and complications. In cancer patients, neuropathic pain may be caused by tumor compression and/or nerve damage due to treatments such as chemotherapy, radiotherapy and surgery. Which is most commonly caused by tumors compressing the spinal nerve roots. About 20 % of cancer pain is estimated to be neuropathic in nature, although mixed pattern of pain can also occur, which is also considered to be a neurological factor [78].

#### 2.4.1 Mechanisms of formation of cancer pain

**Fig. 5:**
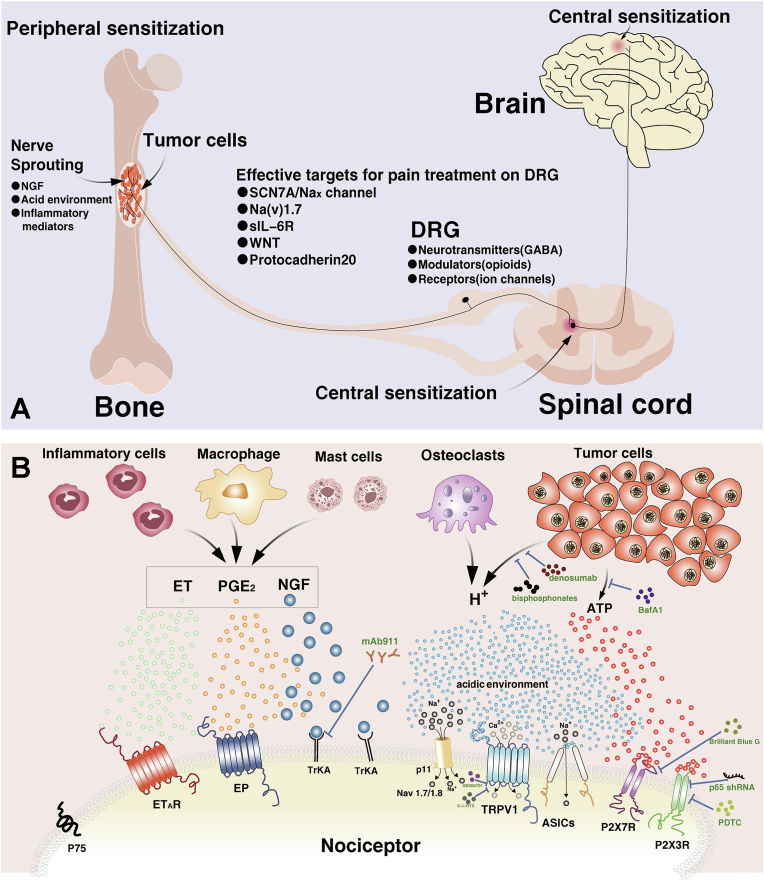
**A.** Shows the mechanism of cancer-induced bone pain. **B.** Representation of the roles of the acidic environment and inflammatory response related pathway in the mechanism of cancer-induced bone pain. Reproduced with permission [209]. Copyright 2021, Elsevier.

Current research indicates several key physiopathological mechanisms involving in cancer pain include the following: 1) Peripheral and central sensitisation: peripheral and central sensitisation are the main mechanisms in the pathogenesis of chronic pain, including cancer pain. Peripheral sensitisation refers to an increased sensitivity of the peripheral nervous system (e.g. sensory nerve endings) to pain signals in response to painful stimuli, whereas central sensitisation refers to the increase in the sensitivity of the central nervous system (e.g. the spinal cord and the brain) to the processing of pain signals. processing becomes more sensitive. Central sensitisation may result in the amplification of pain signals, causing even mild stimuli to be intensely painful (Fig. 5) [79]. 2) Nerve growth factor (NGF): Enhanced expression of NGF and its receptor TrkA has been found in pancreatic cancer, which may promote perineural invasion of the tumor and may be associated with increased pain in patients with pancreatic cancer [80]. Furthermore, it has been found that antibody treatment against NGF can reduce neuropathic pain or inflammatory pain [81]. 3) Acidic environment: Acidification of the tumor microenvironment may exacerbate pain by affecting the function of ion channels. Masahiro Hiasa et al. simulated multiple myeloma (MM) induced severe bone pain (MMBP) by implanting JJN3 cell (a kind of human multiple myeloma cells) into the tibia of a mouse model. We found that osteoclasts and MM act together to induce an acidic bone microenvironment, thereby triggering MMBP through ASIC3 activation of sensory neurons and suggested that targeting ASIC3 on neurons and the acidic bone microenvironment induced by multiple myeloma could be a strategy to alleviate MMBP in patients [82]; 4) Tumor-derived injurious mediators: Cytokines such as tumor necrosis factor-alpha (TNF-α) and interleukin-6 (IL-6) play a prominent role in cancer pain. Increased expression of TNF-α and IL-6 can be observed in different animal models of cancer pain, which in turn produce persistent cancer pain and associated inflammation, and inhibition of TNF-α and IL-6 signaling reduced nociception.

#### 2.4.2 Treatment of cancer pain

Opioids have been the mainstay of treatment for cancer-related pain for decades and are typically administered orally [83]. Concomitantly, opioid painkillers can also lead to a series of side effects in patients, such as gastrointestinal adverse reactions, neurological symptoms and other problems, requiring careful management by healthcare professionals [84]. Most side effects are manageable, and acute or serious adverse reactions are relatively rare. Opioid-induced gastrointestinal dysfunction encompasses a range of adverse effects linked to opioid therapy, comprising constipation, gastroesophageal reflux disease, nausea and vomiting, bloating, and abdominal discomfort. Nausea and vomiting from opioids occur in up to 40 % of patients with cancer who have not previously vomited, but they typically diminish after a few days or weeks of treatment. In severe cases, patients may require oral antiemetic medication to relieve the symptoms. Constipation is mainly characterized by reduced frequency of defecation, straining to pass a bowel movement, a feeling of incomplete emptying of the rectum, or a hardening of the consistency of the stool [85]. Some patients may also exhibit transient mild drowsiness at the beginning of the medication, and in severe cases, uncommon neuropsychiatric symptoms such as agitation or delirium may occur [83]. Adjunctive therapies, such as tricyclic antidepressants (TCAs), 5-hydroxytryptamine and norepinephrine reuptake hibitors (SNRIs), and anticonvulsants, are generally effective in treating neuropathic pain, but can also cause side effects like dizziness, drowsiness, and gastrointestinal symptoms, all of which may affect patient compliance with treatment [86]. Considering the side effects of pharmacological treatments and the limitations of their application (e.g., in patients with renal insufficiency), non-pharmacological treatments such as interventional and minimally invasive procedures have also been widely used in the treatment of cancer pain. These procedures include epidural analgesia, intrathecal drug administration, nerve block, ablative surgery, and palliative radiotherapy, etc. Although these local or targeted therapies can provide effective relief for difficult-to-treat or untreatable cancer pain, they have limitations. Their effects are often temporary, lasting from days to weeks, and rarely months. Nerve blocks, while initially offering excellent pain relief, may carry risks and potential side effects, such as significant impairment of limb movement when ablating nerves with mixed sensory and motor function [87]. Many clinical trials are currently exploring better approaches for cancer pain management, with Table 1 from 2023 onwards. In the future, we need continued research and exploration of new therapeutic modalities are needed to improve pain management outcomes in cancer patients. Furthermore, achieving targeted and individualised pain management programs requires further investigation and development of new treatment strategies.

**Table 1:** Clinical trials of cancer pain treatments.

NCT Number	Current Status	Conditions	Intervention	Phases	Location	Institue
NCT05088876	Receuiting	Pain	Paracetamol	IV	Switzerland	Insel Gruppe AG, University Hospital Bern
NCT05898880	Active, not recruiting	Cancer pain	Acupressure	NA	No location data	Mersin University
NCT05726929	Recruiting	Breast Cancer	Osteopathic treatment	NA	Toulouse, France	Institut Toulousain d'Ostéopathie
NCT05257876	Completed	Breast Cancer	Breathing exercise	NA	Luzhou, Sichuan, China	Charles Darwin University
NCT05753046	Enrolling by invitation	Prostate Cancer	TAP Block	IV	Royal Oak, Michigan, United States	William Beaumont Hospitals
NCT05842044	Recruiting	Kidney Cancer	Non-steroidal anti-inflammatory drugs	II	Miami, Florida, United States	University of Miami
NCT05746429	Recruiting	Bone Sarcoma	Cognitive Behavior Therapy plus Transcranial Direct Current Stimulation	NA	Memphis, Tennessee, United States	St. Jude Children's Research Hospital
NCT05763667	Enrolling by invitation	Gynecologic Cancer	TAP plus Liposomal bupivacaine	III	Boston, Massachusetts, United States	Tufts Medical Center
NCT05730972	Active, not recruiting	Cancer Pain	Transcutaneous electrical acupoint stimulation	NA	Location not provided	The Third Affiliated hospital of Zhejiang Chinese Medical University
NCT06143020	completed	Breast Cancer	Erector spinae plane block	NA	Shanghai, Shanghai, China	Fudan University
NCT04808531	Active, not recruiting	Cancer Related Pain	NanaBis™	III	Location not provided	Medlab Clinical
NCT05993273	Recruiting	Lung Cancer	Cryoanalgesia, Erector Spinae Plane Block or Epidural Catheter	NA	Padua, Italy	University Hospital Padova
NCT06115330	Completed	Gynecologycal Cancer	Battlefield acupuncture	NA	Indonesia	Indonesia University
NCT06073496	Completed	Gynecologycal Cancer	Electroacupuncture	NA	Indonesia	Indonesia University
NCT05317026	Recruiting	Spine Metastases	Vertebroplasty plus Stereotactic Body Radiation Therapy	NA	Montréal, Quebec, Canada	Centre hospitalier de l'Université de Montréal (CHUM)
NCT05419518	Recruiting	Neoplasm Metastases	External beam radiation	II	United States	Rutgers, The State University of New Jersey
NCT05852002	Completed	Advanced Lung Cancer	35 kDa hyaluronan fragment	NA	Ulaanbaatar, Mongolia	Nakhia Impex LLC
NCT06121102	Recruiting	Oropharyngeal Cancer	Supervoltage pulsed radiofrequency glossopharyngeal nerve block	NA	Cairo, Egypt	National Cancer Institute, Egypt
NCT05494502	Recruiting	Breast Cancer	Erector spinae plane block	NA	Beijing, Beijing, China (2)	Peking University First Hospital
NCT06160323	Recruiting	Pancreatic Cancer Non-resectable	EUS-guided celiac ganglion neurolysis/celiac Plexus neurolysis	NA	Hong Kong, Hong Kong	Chinese University of Hong Kong
NCT06017895	Recruiting	Nasopharyngeal carcinoma	Doxepin solution	NA	Guangzhou, Guangdong, China	Nanfang Hospital, Southern Medical University
NCT06228768	Recruiting	Postmenopausal|Breast Cancer	Acupressure	NA	University of Michigan Rogel Cancer Center	Ann Arbor, Michigan, United States
NCT05771103	Recruiting	Post-mastectomy Pain Syndrome	Thermal Radiofrequency neurolysis of Stellate ganglion	NA	Cairo, Egypt	National Cancer Institute, Egypt
NCT06077487	Active, not recruiting	Pancreatic Ductal Adenocarcinoma	Ketamine	IV	San Francisco, California, United States	Brian Anderson, MD
NCT06242587	Active, not recruiting	Coccygeal Body Tumor	Pericoccygeal nerve block	NA	Ankara, Çankaya, Turkey	Diskapi Teaching and Research Hospital
NCT05841186	Active, not recruiting	Breast Cancer	Timing of pegfilgrastim administration	II	Guangzhou, Guangdong, China	uangdong Provincial People's Hospital
NCT06070363	Completed	Gynecologic Cancer	Manual Acupuncture	NA	Indonesia	Indonesia University
NCT05870241	Completed	Post Mastectomy Lymphedema	Ga-As Laser/Microcurrent Electrical Stimulation	NA	Giza, Egypt	Cairo University
NCT06223659	Recruiting	Skin cancers	Eutectic Mixture of Local Anesthetics	II	Columbus, Ohio, United States	Ohio State University Comprehensive Cancer Center
NCT06065449	Recruiting	Bone Metastases	Radiation Therapy	III	Houston, Texas, United States	Houston, Texas, United States
NCT05935059	Recruiting	Post-mastectomy Pain Syndrome	Pregabalin/Tianeptine	NA	Cairo, Egypt	National Cancer Institute, Egypt
NCT06008899	Active, not recruiting	Cancer-related anal or perineal pain	US assisted caudal epidural pulsed radiofrequency	NA	Assuit, Egypt	Assiut University
NCT06341270	Active, not recruiting	Liver Cancer	Transcutaneous electrical acupoint stimulation	NA	Chengdu, Sichuan, China	West China Hospital
NCT06331793	Recruiting	Female Breast Cancer	Active AlgoCare	NA	Milan, Italy	European Institute of Oncology
NCT05670561	Recruiting	Liver Cancer	Esketamine/Sufentanil	IV	Chongqing, Chongqing, China	The Second Affiliated Hospital of Chongqing Medical University
NCT05878405	Recruiting	Malignant tumor	Methylene Blue Oral Rinse	III	Rochester, Minnesota, United States	Mayo Clinic
NCT05597878	Recruiting	Prostate Cancer	Opioid-Free Pain Control Regimen	II/III	Winston-Salem, North Carolina, United States	Wake Forest University Health Sciences
NCT05315011	Recruiting	Breast Cancer	Whole-body cryotherapy	NA	France	University Hospital, Montpellier
NCT06265077	Active, not recruiting	Breast Cancer	Famotidine and loratadine	I/II	Location not provided	Noha Mansour
NCT06240377	Active, not recruiting	Pain Cancer	tDCS/NMP	NA	Santa Cruz De Tenerife, Spain	Universidad Europea de Canarias

TAPB: transversus abdominis plane block; tDCS: Transcranial direct current stimulation; NMP: Ultrasound-guided percutaneous neuromodulation.

### 2.5 Side effects of cancer drug therapy---immunotherapy

Traditional anti-tumor treatments, such as surgery, radiotherapy, and chemotherapy, which, despite being widely used, often have poor therapeutic outcomes and are accompanied by significant side effects [88]. However, in recent years, with the progress of research in tumor immunotherapy, such as dendritic cell vaccine, adoptive cell therapy, small molecule targeted drug therapy and anti-tumor monoclonal antibody, especially targeted immune checkpoint therapy, tumor immunotherapy, as a fourth therapeutic modality in addition to traditional therapies, has exerted a great influence in the comprehensive treatment of tumors [89]. Tumor immunotherapy is characterised by high specificity and low side effects compared to conventional treatments [90]. A significant advancement in tumor immunotherapy is the development and clinical use of immune checkpoint inhibitors (ICIs), particularly inhibitors such as cytotoxic T lymphocyte-associated molecule-4 (CTLA-4), programmed death 1 (PD-1), and PD ligand 1 (PD-L1). ICIs have shown promising results in various tumor types. However, not all cancer patients benefit from immunosuppressive therapy, and more research is needed to overcome resistance in this treatment approach [91].

#### 2.5.1 Mechanisms of tumour immunotherapy

**Fig. 6:**
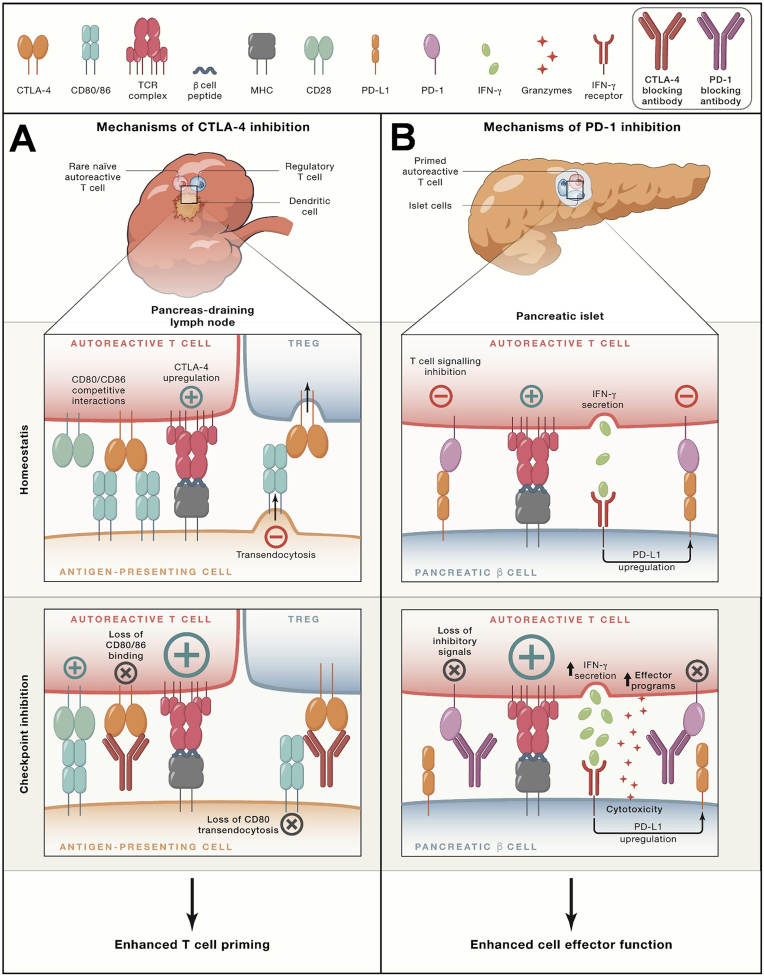
Modulation of T cell function by antibodies targeting the inhibitory CTLA-4 and PD-1 receptors. **A.** Biology of CTLA-4 receptor. The CTLA-4 inhibitory and CD28 costimulatory receptors bind to the same ligands (CD80/CD86) on antigen presenting cells in lymph nodes. CTLA-4 binds these ligands with higher affinity, thereby reducing ligand availability for CD28. CTLA-4 is stored in an intracellular compartment and transported to the cell surface following initial T cell activation, thereby serving as a negative feedback mechanism. Antibody-mediated inhibition of CTLA-4 function enhances T cell priming by making more CD80/CD86 ligands available for the CD28 costimulatory receptor. Also, the antibody prevents removal of CD80/86 from antigen presenting cells by Tregs. **B**. Biology of the PD-1 receptor. PD-1 expression is induced by T cell activation, thereby providing an inhibitory signal that constrains T cell function. IFNγ secreted by activated T cells induces expression of PD-L1 on target cells (such as pancreatic β cells). This pathway inhibits autoimmunity and immunopathology. Antibody-mediated blockade of this pathway enhances T cell activation, resulting in greater cytotoxicity and release of pro-inflammatory cytokines [8]. Copyright 2021, Cell.

**Fig. 7:**
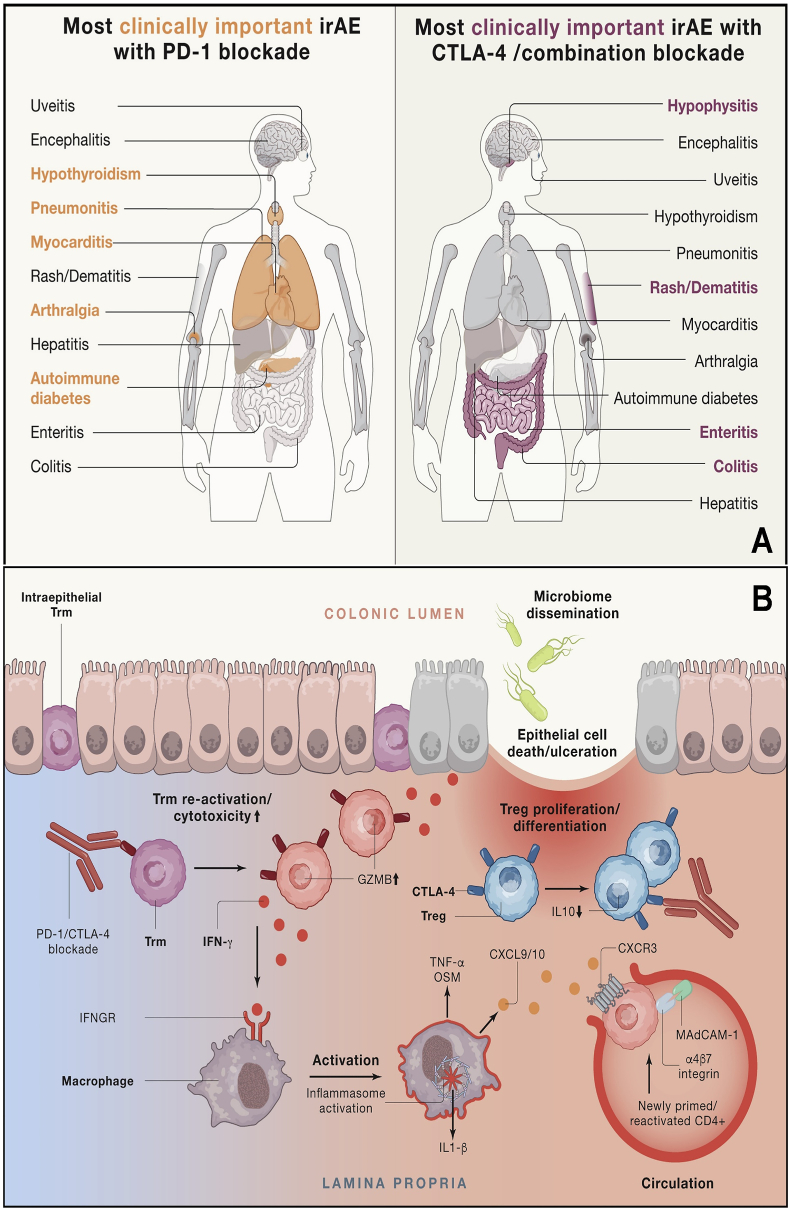
**A.** Organs frequently affected by inflammatory toxicities of checkpoint blockade. **B.** Inflammatory pathways contributing to colon inflammation following checkpoint blockade [210]. Copyright 2021, Cell.

The exact pathophysiology underlying immune-related adverse events is not fully understood. However, current understanding suggests that these adverse events may be associated with the role of immune checkpoints in regulating immune homeostasis [92]. Various biomarkers have been suggested to be associated with ICI-induced irAE. While no biomarkers have been recognised as reliable predictors to date, some have shown clinical value and have been evaluated as pharmacodynamic markers after treatment [93]. Close monitoring and early intervention in patient treatment are crucial to minimize the incidence of irAEs. T cells play a central role in checkpoint blockade therapy by recognizing peptide-MHC complexes on tumor cells through their receptors. Co-stimulatory signals such as CD28 contribute to the full activation of T cells, while co-inhibitory receptors like CTLA-4 and PD-1 suppress this activation (Fig. 6). A clinical trial involving metastatic prostate cancer patients revealed that the clonal expansion of CD8 T-cells could serve as an early biomarker for predicting adverse effects induced by ipilimumab. However, in two clinical trials involving tremelimumab-treated stage III or IV unresectable melanoma patients, investigations uncovered a whole blood mRNA marker predictive of clinical outcomes in individuals subjected to immune checkpoint blockade therapy, thus addressing the need for predictive biomarkers associated with treatment-related irAEs [94]. When managing patients receiving multiple immune checkpoint inhibitors, such as PD-1/PD-L1 and CTLA-4 inhibitors, early detection of specific B-cell alterations can serve as a predictor for autoimmune reactions. This research has facilitated the identification of patients at the outset of treatment who are susceptible to autoimmune responses at the beginning of treatment, enabling timely interventions to mitigate adverse events and optimize treatment protocols [95]. Colitis represents the predominant adverse reaction to anti-CTLA-4 immune checkpoint therapy, where biomarkers to forecast severe diarrhoea associated with this treatment are still lacking (Fig. 7B). The increasing number of patients on immunotherapy and the impact and patients’ IrAEs have become a major obstacle to the prospects for the use of ICIs, and there is still a need to actively explore ways of mitigating the toxicity of ICIs to maximise control of immune dysregulation and minimize the risk of opportunistic infections.

#### 2.5.2 Adverse effects of tumour immunotherapy

Despite its enormous potential, immunotherapy is accompanied by immune-related adverse events (irAE) that can limit its effectiveness. Clinical trials related to irAE in the last year are shown in Table 2 [96]. These adverse effects may result in treatment interruptions and cessation, and in rare instances, they may even be life-threatening [92]. Compared to adverse events induced by chemotherapy and targeted therapy, the severity and timing of irAEs are unpredictable, and various organs throughout the body may be involved [93]. While immune-related adverse events can affect any organ system, the gastrointestinal tract, endocrine glands, skin, and liver are most commonly involved (Fig. 7A) [97]. Specifically, Patients treated with monoclonal antibodies against PD-1 or its ligands are at an increased risk of experiencing hypothyroidism, hepatotoxicity, and pneumonia. Colitis has emerged as a leading cause of mortality in individuals treated with anti-CTLA-4 antibodies [98]. These events can manifest at any time, even after discontinuation of immune checkpoint blockade therapy, and their frequency may fluctuate. Nevertheless, most research indicates that prolonged treatment does not increase the cumulative occurrence of immune-related adverse events.

**Table 2:** Clinical trials on immune-related adverse reactions.

NCT Number	Current Status	Conditions	Immunotherapy types	Categories	Phases	Location	Institue
NCT03655002	Active, not recruiting	Hepatocellular Carcinoma	Cytokine therapy/ICIs	Immunomodulators/PD-1 Inhibitors	I	United States	City of Hope Medical Center
NCT03812562	Terminated	Hepatocellular Carcinoma	ICIs	PD-1 Inhibitors	I	Chicago, Illinois, United States	Northwestern University
NCT03439891	Active, not recruiting	Hepatocellular Carcinoma	ICIs	PD-1 Inhibitors	II	United States	Robin Kate Kelley
NCT03222076	Completed	Hepatocellular Carcinoma	ICIs	PD-1 Inhibitors	II	Houston, Texas, United States	M.D. Anderson Cancer Center
NCT06045286	Recruiting	Colorectal Liver Metastases	ICIs	PD-1 Inhibitors	I	Nanjing, Jiangsu, China	Jiangsu Cancer Institute & Hospital
NCT05168163	Recruiting	Hepatocellular Carcinoma	ICIs	PD-L1 Inhibitors	II	United States	Academic and Community Cancer Research United
NCT03472586	Active, not recruiting	Metastatic Malignant Neoplasm in the Liver/Metastatic Uveal Melanoma	ICIs	PD-1 Inhibitors	II	Philadelphia, Pennsylvania, United States	Sidney Kimmel Cancer Center at Thomas Jefferson University
NCT05063565	Recruiting	Hepatocellular Carcinoma	ICIs	PD-L1 Inhibitors/CTLA-4 inhibitor	II	United States	Boston Scientific Corporation
NCT05622071	Recruiting	Hepatocellular Carcinoma	ICIs	PD-1 Inhibitors	II	France	UNICANCER
NCT04777708	Terminated	Advanced Hepatocellular Carcinoma	ICIs	PD-1 Inhibitors	I	Los Angeles, California, United States	Jonsson Comprehensive Cancer Center
NCT03680508	Recruiting	Adult Primary Liver Cancer	ICIs	PD-1 Inhibitors	II	United States	University of Hawaii
NCT04021043	Active, not recruiting	Advanced Malignant Solid Neoplasm	ICIs	PD-1 Inhibitors	I/II	Houston, Texas, United States	M.D. Anderson Cancer Center
NCT05211323	Recruiting	Hepatocellular Carcinoma	ICIs	PD-L1 Inhibitors	II	United States	National Cancer Institute (NCI)
NCT05194293	Recruiting	Hepatocellular Carcinoma	ICIs	PD-L1 Inhibitors	II	United States	Academic and Community Cancer Research United
NCT05169957	Recruiting	Melanoma	ICIs	PD-1 Inhibitors	I	Ann Arbor, Michigan, United States	University of Michigan Rogel Cancer Center
NCT04828486	Recruiting	Advanced Hepatocellular Carcinoma	ICIs	PD-1 Inhibitors	II	Rochester, Minnesota, United States	Mayo Clinic
NCT03436563	Active, not recruiting	Colon Cancer/Rectal Cancer	ICIs	PD-L1 Inhibitors/Immunomodulators	I/II	Houston, Texas, United States	M.D. Anderson Cancer Center
NCT04631731	Recruiting	Malignant Solid Neoplasm	ICIs	PD-1/L1 inhibitor plus CTLA-4 inhibitor	I/II	Sydney, New South Wales, Australia (2)	Western Sydney Local Health District
NCT06239220	Recruiting	Head and Neck Squamous Cell Carcinoma	Immunocellular therapy	CAR-T Cell Therapy	II	Boston, Massachusetts, United States (2)	Glenn J. Hanna
NCT03050060	Terminated	Malignant Solid Neoplasm	ICIs	PD-L1 Inhibitors/PD-1 Inhibitors	II	Seattle, Washington, United States	University of Washington
NCT04562129	Recruiting	Melanoma	Cytokine therapy/ICIs	Cytokine therapy/PD-1 Inhibitors	II	Tampa, Florida, United States	H. Lee Moffitt Cancer Center and Research Institute
NCT02978625	Active, not recruiting	Skin Carcinoma	vaccine therapy/ICIs	vaccine therapy/PD-1 Inhibitors	II	United States	National Cancer Institute (NCI)
NCT05393713	Active, not recruiting	Melanoma	ICIs	PD-L1 Inhibitors	II	Rochester, Minnesota, United States	Mayo Clinic
NCT03071406	Active, not recruiting	Merkel Cell Carcinoma/Skin Cancer	ICIs	PD-1 Inhibitors	II	United States	H. Lee Moffitt Cancer Center and Research Institute
NCT04387084	Recruiting	Skin Neoplasm	ICIs	PD-L1 Inhibitors/PD-1 Inhibitors	II	Los Angeles, California, United States (2)	University of Southern California
NCT05878964	Recruiting	Malignant Solid Neoplasm	ICIs	PD-1/PD-L1/CTLA-4 or Cyclin-Dependent Kinase (CDK) Inhibitors	II	Roma, Italy	Fondazione Policlinico Universitario Agostino Gemelli IRCCS
NCT04315701	Recruiting	Skin Cancer	ICIs	PD-1 Inhibitors	II	United States	University of Southern California
NCT01702792	Terminated	Glioblastoma	Vaccine therapy	Vaccine therapy	II	Location not provided	Dartmouth-Hitchcock Medical Center
NCT03944941	Active, not recruiting	Skin Carcinoma/Metastatic Skin Cancer	ICIs	PD-L1 Inhibitors	II	United States	Alliance for Clinical Trials in Oncology
NCT04204837	Recruiting	Squamous Cell Carcinoma of the Skin	ICIs	PD-1 Inhibitors/CTLA-4 inhibitor	II	Austria	Salzburger Landeskliniken
NCT05335928	Recruiting	Myocarditis Acute/Cancer	Cytokine therapy	immunomodulators	III	United States	Massachusetts General Hospital
NCT05127824	Recruiting	Carcinoma, Renal Cell	Vaccine therapy	Dendritic cell vaccine/Tumor-Associated Antigen	II	Pittsburgh, Pennsylvania, United States	Jodi Maranchie
NCT05927142	Recruiting	Metastatic Pancreatic Cancer	Cytokine therapy/ICIs	PD-L1 Inhibitors	I/II	Rotterdam, Zuid-Holland, Netherlands	Joachim Aerts, MD PhD
NCT04991870	Recruiting	Gliosarcoma	DDSs	Engineered Natural Killer Cells	I	Houston, Texas, United States	M.D. Anderson Cancer Center
NCT04690855	Terminated	Breast Cancer	ICIs	PD-L1 Inhibitors	II	United States	Mylin A. Torres, MD
NCT05288569	Recruiting	Lung Cancer	ICIs	anti-PD-1/anti-PD-L1 immunotherapy	NA	Beijing, China	Peking Union Medical College Hospital
NCT04552704	Terminated	Advanced Malignant Solid Neoplasm	Cytokine therapy	Immunomodulators	I/II	Sacramento, California, United States	Tianhong Li
NCT04375228	Recruiting	Advanced Solid Tumor	ICIs	PD-1 Inhibitors	II	United States	Columbia University
NCT05714371	Completed	Melanoma	ICIs	PD-1 Inhibitors/PD-1inhibitor plus CTLA-4 inhibitor	NA	East Hanover, New Jersey, United States	Novartis Pharmaceuticals
NCT05717712	Recruiting	Oncolytic Virus|Diffuse Intrinsic Pontine Glioma	Cytokine therapy	immunomodulators	I	Beijing, Beijing, China	Capital Medical University
NCT06229067	Active, not recruiting	Breast Cancer	ICIs	PD-L1 Inhibitors	II	Location not provided	Cancer Institute and Hospital, Chinese Academy of Medical Sciences
NCT05914389	Recruiting	Colon Neoplasm	ICIs	Anti-PD-L1 Monoclonal Antibody	II	Hangzhou, Zhejiang, China	Second Affiliated Hospital, School of Medicine, Zhejiang University
NCT06269133	Active, not recruiting	Advanced Non-small Cell Lung Cancer	ICIs	PD-1 Inhibitors	NA	Tarrytown, New York, United States	Regeneron Pharmaceuticals

The mechanisms behind these adverse reactions involve the disruption of the body's immune tolerance, the promotion of existing autoimmune development, cross-reactive immune responses, the release of a large amount of cytokines by activated T cells, and off-target effects. irAEs can occur at different points after treatment, ranging from a few days to several months, with varying degrees of severity, some of which may require discontinuation of immune checkpoint inhibitor therapy and treatment with high-dose corticosteroids. Therefore, for patients undergoing cancer immunotherapy, it is necessary to closely monitor these potential adverse reactions and take appropriate management measures in a timely manner to ensure patient safety and the effectiveness of the treatment.

The treatment of malignant tumors is a comprehensive and multidisciplinary process that requires attention not only to the therapeutic effects of the patient's primary tumor, but also to the occurrence of complications. Effective management of complications is crucial in improving the overall treatment outcomes and quality of life for cancer patients. Therefore, rational management of complications during the treatment of malignant tumors is crucial. This includes the prevention of complications, timely interventions to manage them, and a holistic approach to the patient's overall condition, which can help to improve the effectiveness of treatment and alleviate patient suffering, as well as provide significant benefits to the patient's long-term survival and quality of life.

## 3 Application of drug delivery in the treatment of cancer complications

### 3.1 Drug delivery systems

Drug delivery is known as the last kilometre of drug development and is currently a key technological challenge in biopharmaceuticals. The overarching objective of DDSs is to precisely deliver therapeutics to targeted sites at appropriate times, thereby enhancing efficacy while mitigating adverse effects. DDSs have several core benefits. 1) Controlled drug release timing and dosage: by slowing down the rate of drug release and maintaining the effective concentration for a longer period, it reduces the frequency of drug administration and related toxic side effects [99]. By developing smart responsive materials that can respond to specific stimuli in the tumor microenvironment, such as changes in pH, enzyme activity, or temperature, drugs can be precisely released when needed. Furthermore, by modifying targeting ligands and molecules, DDS can be designed to release drugs only when they reach the target cells or tissues, reducing the impact on healthy tissues. In terms of dosage control, researchers regulate the release kinetics of drugs by precisely designing the physical and chemical properties of DDS, including using multilayer or multi-stage release systems to achieve a combination of initial rapid release and subsequent slow, sustained release, in order to maintain drug concentrations within the therapeutic window. Additionally, the application of 3D printing and microfluidic technologies makes the manufacturing of DDS more precise, further controlling the spatiotemporal characteristics of drug release. 2) Reduce adverse drug reactions: the drug is precisely directed to the lesion site and specifically aggregated in the target cells and tissues to mitigate the damage to healthy tissues for more precise treatment [100]. Using strategies such as biocompatible materials, surface modifications, targeting ligands, and smart responsive designs can effectively reduce the adverse effects of drugs on non-cancerous tissues. By selecting materials like polysaccharides [101], polyphenylalanine [102], graphene derivatives [103]and metal-organic frameworks, combined with polyethylene glycol (PEG) surface coating, the biocompatibility and targeting of DDSs can be enhanced, reducing immune and foreign body reactions. Utilizing targeting ligands such as antibodies, peptides, or small molecules ensures that DDSs specifically bind to certain receptors on cancer cells or in the tumor microenvironment, minimizing distribution to normal tissues. Additionally, by responding to stimuli in the tumor microenvironment to achieve precise drug release, these synergistic strategies not only improve therapeutic outcomes but also enhance the quality of life for patients. Further preclinical evaluations and personalized medicine will optimize the design of DDSs to ensure the safety and efficacy of drug delivery. 3) Enhance drug stability: improve the solubility and dissolution rates of drugs with low solubility in vivo, protect the drugs from phagocyte clearance and various enzyme attacks in vivo, improve the stability of the drugs in vivo, and also regulate the metabolic rate of the drugs a), [104]. Drug delivery technologies have markedly improved the solubility of drugs through the implementation of a suite of innovative strategies. These methodologies not only optimize the solubility profiles of pharmaceuticals but also facilitate precise modulation of drug release kinetics, augment the bioactivity of therapeutic agents, and fine-tune their pharmacokinetic attributes. Within this spectrum of advancements, nanotechnology emerges as a particularly impactful tool, significantly enhancing drug solubility and modulating their in vivo distribution, thereby augmenting the precision of drug targeting. This enhancement in targeting precision translates into improved therapeutic outcomes and a concurrent reduction in the incidence of adverse reactions. Nanometerials, capitalizing on their distinctive size-dependent effects and inherent properties, are pivotal in amplifying the efficacy of drug delivery to specific targets and mitigating off-target effects. The ongoing evolution and clinical application of these technologies have substantially ameliorated the therapeutic efficacy of drugs with poor solubility profiles, concurrently elevating the standard of patient care. 4) Improved drug absorption: increase the efficiency of drug absorption through the intestinal mucosa, skin, and other routes. For example, alkyl polyglycoside (APG) has become an ideal permeation enhancer in drug delivery systems due to its efficient transdermal performance, good safety, and biodegradability. APG shows great potential in improving the ability of drugs to penetrate biological membranes. PEG modification can reduce the interaction between drugs and biological barriers (such as cell membranes, mucus layers), thereby enhancing the penetration ability of drugs [105]. In addition, utilizing nanoparticles can break through biological barriers, such as the blood-brain barrier. Research has found that by precisely designing the surfactants and receptors on the surface of nanoparticles, the crossing of drugs through the blood-brain barrier can be facilitated, enhancing the therapeutic effect on the damaged brain [106]. Facilitate the penetration of drugs across specific biological barriers like the blood-brain barrier and cell membranes, enhancing drug absorption bioavailability, and ultimately enhancing therapeutic efficacy [107]. The development of modern drug delivery technology dates back to the introduction of Spansule® slow-release capsule technology in 1952 [108]. Since then, the new drug delivery technologies have been widely used in various diseases, including cardiovascular, cerebrovascular, diabetic, and metabolic diseases, as well as malignant tumors [109]. Currently, various types of delivery systems are based on chemical materials such as lipid nanoparticles, liposomes, and lipid micelles. Other systems include exosome delivery systems based on cellular vesicles, erythrocyte, and platelet delivery systems using living cells, as well as novel DDSs based on micro- and nanorobotics that have emerged in recent years have been developed and applied [110].

The application of drug delivery systems in the field of oncology is of great significance. DDSs are widely used in emerging therapeutic strategies such as combination therapy and immunotherapy [111]. Nano drug delivery systems utilize their nanoscale carrier properties to achieve precise targeting based on tumor microenvironment characteristics (e.g. receptor overexpression or abnormal vascular structure), enhancing drug accumulation in tumor tissues. For example, liposomes and micelles can enhance drug solubility and stability by encapsulating them into microparticles, while polymer carriers can modulate the release rate and targeting of drugs. Moreover, DDSs can also be employed in gene therapy to deliver gene vectors precisely into tumor cells for targeted gene therapy [112]. Overall, DDSs facilitate precise drug delivery, enabling direct action on diseased tissues while minimizing damage to healthy tissues, thus improving therapeutic efficacy while alleviating patients’ pain, and precisely controlling the time and location of drug release. These technologies not only provide the feasibility of personalized treatment, but also greatly optimize the quality of life of patients.

### 3.2 Cancer-associated thrombosis and drug delivery systems

Current drug dosing regimens are insufficient to prevent effective thrombosis, and more targeted drug delivery systems have attracted much attention by showing potential in preclinical studies [113].

#### 3.2.1 Nanodrug delivery systems

Nano Drug Delivery Systems (NDDS) are technologies that utilize nanoscale materials as drug carriers, capable of improving the pharmacokinetic behavior of drugs, precisely delivering active pharmaceutical ingredients to the lesion, and controlling drug release. These systems have shown tremendous potential in the field of medicine and have become a hot topic of research. NDDS, through its unique size effects and surface effects, can increase drug solubility, alter the distribution of drugs within the body, and enhance drug targeting, thereby improving therapeutic efficacy and reducing the incidence of adverse reactions. The design developed by Wang et al. represents an innovative approach to achieve targeted and controlled delivery of thrombolytic drugs through a precise delivery strategy, which can complete thrombus targeting and assist thrombolytic drugs to enter into the thrombus within a short time, significantly improving the thrombolytic rate. The nano-assemblies encapsulate the thrombolytic drug in their nanoparticle shell layer, which protects the activity of tissue plasminogen activator (tPA), and the nano-assemblies are guided to the thrombus by a magnetic field. Ultrasound-induced vibrations of air nuclei are utilized to release the drug-loaded particles, allowing them to cross obstacles and achieve thrombolysis within the thrombus. In a mouse venous thrombosis model, compared with traditional tPA injection, the residual thrombus was reduced by 67.5 % when using nanoparticle-shelled microbubbles (MMB-SiO_2_-tPA). Moreover, in the mouse model, low-intensity ultrasound enhanced the efficiency of femoral vein thrombus dissolution. Compared with physiological saline, natural tPA, and SiO_2_-tPA, the thrombus area in mice treated with MMB-SiO_2_-tPA was smaller, and histological analysis showed more complete thrombus dissolution [114]. Professor Wang's team recently developed a smart DNA nanodevice for precise thrombolytic therapy. The team constructed nanocarriers capable of localising thrombi in vivo and releasing tPA based on thrombin concentration through DNA origami technology. This nanodevice can recognise thrombin, a biomarker of blood clots, in complex pathophysiological environments within blood vessels, and differentiate between blood clots and wound clots through logical operations targeting thrombin concentration. This approach significantly improves therapeutic efficacy and reduces coagulation abnormality caused by thrombolytic drugs, realizing intelligent and precise drug delivery, and has been validated in animal models of cerebral stroke, pulmonary embolism, and carotid artery and leg vein thrombosis. The results of this study suggest a method to develop next-generation smart DNA nanodevices by integrating logic gates based on biomarkers of thrombosis sites. The innovation of smart DNA nanodevices lies in their ability to precisely control drug release, which opens up new possibilities for personalized medicine and improving the safety and efficacy of thrombolytic therapy [115].

#### 3.2.2 Platelet drug delivery System

**Fig. 8:**
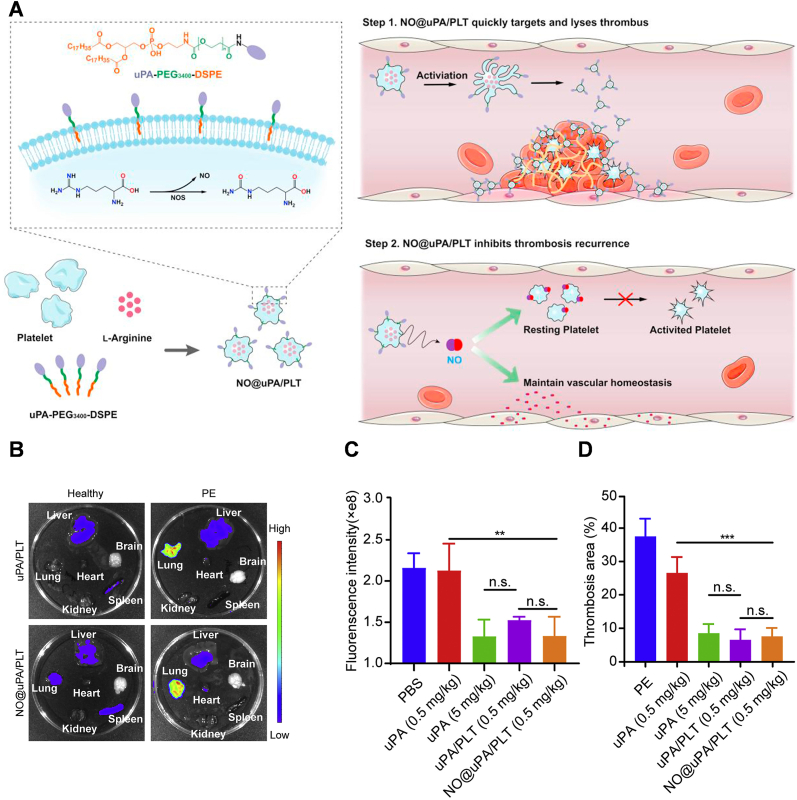
**A.** Illustration of the engineered platelet-based drug delivery platform. The uPA/Arg coloaded platelet delivery system provided a unique combination to achieve targeted delivery of uPA to thrombi, prolong the circulation duration of uPA and prevent the recurrence of thrombi with limited haemorrhagic risk. **B.** Fluorescence images of uPA/PLTs biodistribution and NO@uPA/PLTs biodistribution in the major organs of the PE model and healthy mice. **C.** Fluorescent images of the lung tissues harvested from the mice after different treatments. The pulmonary embolism was labeled with Cy5. **D.** Fluorescence intensity of Cy5 in lung tissues with different treatments (n = 3). Reproduced with permission [211]. Copyright 2022, Elsevier.

**Fig. 9:**
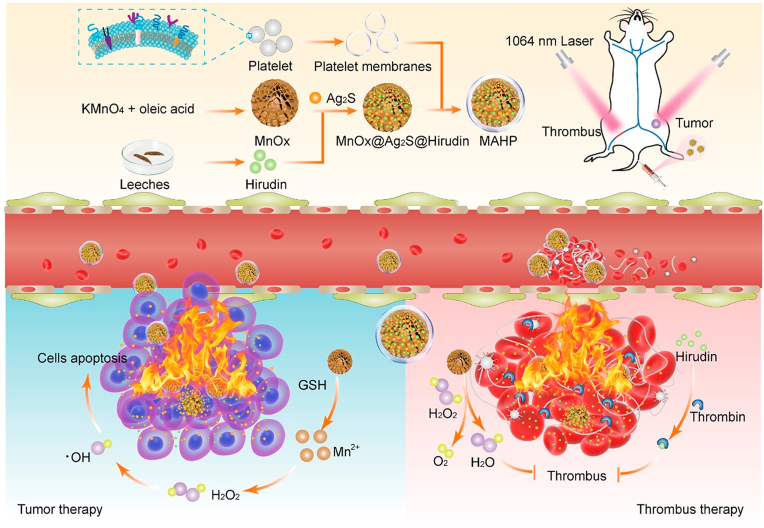
Schematic diagram of MAHP for NIR-II light triggered delivery of hirudin in simultaneous thrombosis therapy and tumor suppression. The released hirudin binds to thrombin at the thrombus site to enable it to lose the coagulation function and combines with the photothermal effect of Ag2S to dissolve the thrombus. MnOx could be decomposed by GSH in the tumor to generate Mn2+ for producing highly toxic •OH through Fenton-type reaction, which integrates with the photothermal effect of Ag2S to induce tumor cell apoptosis. Reproduced with permission [212]. Copyright 2022, American Chemical Society.

The Platelet Drug Delivery System is a technology that utilizes platelets in human blood as drug carriers, offering advantages such as good biocompatibility, long blood circulation time, and strong in vivo targeting capabilities. Furthermore, bionic nanocarriers based on platelet membranes have been developed, harnessing the targeting and precise drug delivery capabilities of platelets. These nanocarriers, derived from platelet membranes, offer unique advantages such as avoiding potential drug side effects and exhibiting strong targeting abilities. They hold promise as powerful tools for antithrombotic therapy [116]. Utilizing experimental validation in murine models, Wang et al. engineered a platelet-derived delivery platform (NO@uPA/PLTs) that comprised urokinase (uPA) and arginine (Arg) for precise thrombolysis and prevention of re-embolism. This platform exhibited superior efficacy in preventing thrombus recurrence in a murine model with minimal hemorrhagic risk, offering a promising avenue for rapid thrombolysis and effective inhibition of post-treatment re-embolism (Fig. 8) [117]. Zhao et al. also developed a novel antithrombotic therapy strategy based on platelet delivery for the first time. They utilized platelet membrane as the “shell” and high photothermal conversion efficiency (PCE) polymers as the “ammunition”. These platelet-based nanocarriers actively targeted the thrombus site, and the photothermal effect accelerated clot dissolution and improved thrombolytic effect. This approach reduced bleeding and side effects associated with existing therapies, overcoming their limitations. In the rat femoral vein thrombosis model, compared with monotherapy of light therapy or drug treatment alone, this combined therapy achieved up to 90 % thrombolytic effect. It provides a new direction for remote, precise, and controllable sustained thrombolysis, aligning with the trend of translating nanomedicines into clinical practice [118]. Thus, it is crucial to address the risk of thromboembolic complications in conjunction with tumor therapy. However, the therapeutic mechanisms and pathological microenvironment of tumors and thrombi are quite different, which makes the development of potent drugs that can eliminate the risk of thrombosis and at the same time treat cancer extremely challenging, and this puts higher demands on the design of anti-cancer drugs. Professor Han's team achieved non-invasive thrombolysis and effective anticoagulation by developing a bionic nano-delivery system for natural hirudin [119]. They also devised a bionic MnOx/Ag2S nanosubstrate platform, modified with platelet membrane (MnOx@Ag2S@MAHP@platelet membrane: MAHP), for prolonged release of anticoagulant drugs for thrombosis and oncological treatment. This MAHP platform facilitates targeted hirudin delivery to the thrombus site and controlled release under near-infrared light irradiation. The platform demonstrated effective thrombus clearance and exhibited tumor growth inhibition, leading to extended survival in mice with thromboembolic complications, achieving dual therapy for both tumor and thrombosis (Fig. 9) [120].

Taken together, the development of novel DDSs for antithrombotic is promising, and is expected to make breakthroughs in targeting and high efficiency, overcoming multidrug resistance, intelligent and controllable release, and combined therapeutic strategies. By achieving precise targeting of drug delivery, reducing side effects, overcoming drug resistance, achieving intelligent release, and combining with other therapeutic means to provide comprehensive treatment for patients with combined tumors or other diseases, drug efficacy can be enhanced, the quality of life of patients can be improved, and even the rate of death can be reduced.

### 3.3 Malignant serous effusion and drug delivery systems

#### 3.3.1 Extracellular vesicle-based systems

Extracellular vesicle-based systems are technologies that leverage naturally secreted nanoscale vesicles from cells as drug delivery vehicles. These vesicles have tissue tropism and can achieve targeted drug delivery through surface molecules such as integrins and glycans, showing great potential in drug delivery applications. Moreover, EVs can be loaded with therapeutic agents using physical or chemical methods, and their circulation time can be extended and targeting improved through additional modification strategies. Therefore, extracellular vesicle systems play a significant role in drug delivery strategies in fields such as cancer treatment. Recently, there has been growing interest in utilizing cell membrane-derived microparticles (MPs) as natural DDSs due to their crucial role in intercellular communication. One emerging biotechnology platform is the use of drug-carrying vesicles based on extracellular vesicles (EVs), known as drug-loaded tumor microparticle immunotherapeutics (DTMI). The novel tumor bio-immunotherapy technology was pioneered and invented by the Chinese researchers. MPE is the first indication for which DTMI has been used. In 2019, Professor Jin's team used tumor cell-derived MPs (TMPs) as carriers to encapsulate chemotherapeutic agents for the treatment of malignant tumors - the drug-carrying vesicle technique for the treatment of tumors and verified that this novel combined tumor treatment modality combines the advantages of targeted therapies, immunotherapies, and biochemotherapeutic technologies, and has the advantages of targeted focus, activation of tumor and activate the anti-tumor function of immunity. The technology involves stripping off the vesicles, the “coat” of the tumor cells, loading the vesicles with minute amounts of chemotherapeutic drugs before delivering the drug-carrying vesicles to the patient's body and precisely directing them to the tumor site, which achieves a low toxicity and side-effects while increasing the efficiency of killing tumor cells. By mimicking the tumor cells' outer layer, the drug-carrying vesicles avoid detection by the tumor and efficiently release the chemotherapeutic drugs near the tumor cell nucleus, resulting in enhanced tumor cell destruction. Additionally, this technology selectively targets tumor stem cells, which are prone to recurrence and metastasis, and helps overcome tumor cell drug resistance. A clinical study involving 11 patients with MPE treated with DTMI showed promising results. Four patients had complete remission, 6 patients had partial remission, and 1 patient had no response. The objective clinical remission rate was 90.91 %. The average time to achieve pleural adhesion was 7 days, of which 4 patients achieved complete remission in only 5 days, and the median survival of 11 patients was 240 days, which demonstrated the safety and efficacy of this technique in treating refractory recurrent malignant pleural effusion [121].

#### 3.3.2 Nanodrug delivery systems

Neutrophils are the predominant cell group in the body that defends against bacterial invasion and flow with the blood circulation. Once an infection occurs in the body, neutrophils leave the blood vessels and enter the site of infection to attack the pathogenic bacteria, and then a large number of neutrophils die at the site of infection to form a yellowish pus. During the long evolutionary process, neutrophils release DNA and histones from their nuclei at the time of death, forming a reticular complex. Due to the high viscosity of DNA, this mesh structure can stick and wrap the surrounding pathogenic bacteria, effectively killing them, thus it is called neutrophil extracellular trap (NET) [122]. Professor Huang's team found that methotrexate (MTX)-packaging, tumor cell–derived microparticles (MTX-MP) can act as a potent immunotherapeutic agent by mobilising and activating neutrophils and triggering neutrophil recruitment. In cancerous pleural fluid, NETs can also be released. These highly adhesive NETs, as effective biomaterials, can attach to damaged blood vessels and prevent intravascular fluid outflow [123]. Clinical studies have shown that MPEs contain a large number of tumor-associated myeloid cells with a pro-tumor phenotype, which impairs anti-tumor immunity [124]. Professor Zhao's team has further developed a liposomal nanoparticle loaded with cyclic dinucleotide (LNP-CDN) to target stimulators activating interferon gene signaling in macrophages and dendritic cells. Administered intrapleurally, these nanoparticles induce significant changes in the transcriptional milieu of MPE, thereby attenuating immune-cold-type MPE in effusions and pleural tumors. When combined with PD-L1 blockade, immunotherapy integrated with LNP-CDN effectively reduces MPE volume, suppresses tumor growth in the pleural cavity and lung tissue, and extends the survival of mice carrying MPE. This highlights the potential of intrathoracic LNP-CDN for MPE immunotherapy in clinical applications [125]. Currently, research on DDSs for the treatment of malignant serous effusion therapy is in a rapid development stage. Advanced DDSs, such as nanotechnology-based targeted delivery systems, not only improve the local efficacy and reduces the systemic toxicity of the drugs, but also helps to overcome drug resistance. These systems offer a promising avenue for precise treatment of malignant serous effusion, and provide more effective and personalized therapeutic solutions for the patients.

### 3.4 Tumor-associated infections and drug delivery

Infections are typically treated by intravenous or oral administration, but most of the antibiotic treatments can lead to gastrointestinal adverse reactions such as gastric upset, vomiting, loss of appetite or diarrhoea, as well as some mild rashes or mild allergic skin reactions, musculoskeletal, hepatic and renal toxicity, and in more severe cases, antibiotics may also cause bacterial dysbiosis with severe watery diarrhoea and so on [126].

#### 3.4.1 Nanodrug delivery systems

**Fig. 10:**
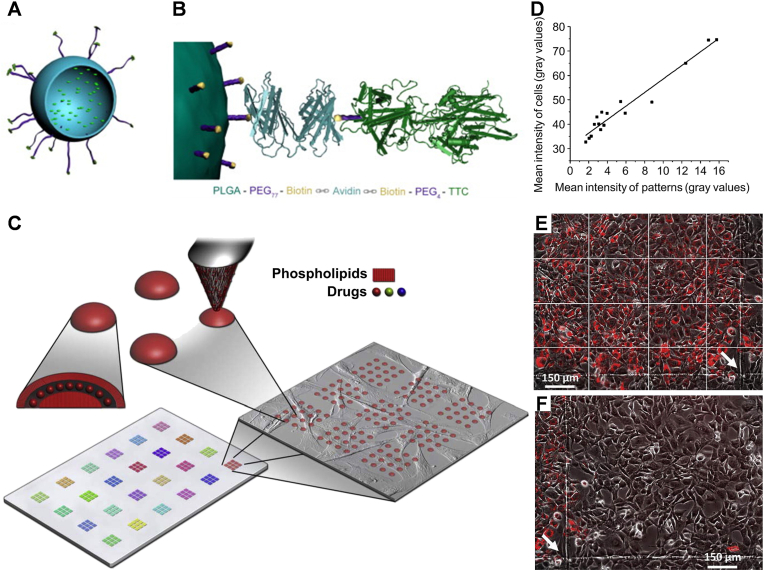
Conceptual schematic (not to scale) illustrating (**A**) the cross-section of a biodegradable polymer nanoparticle that is functionalized with the tetanus toxin C fragment (TTC) and presumed to encapsulate a therapeutic substance,and (**B**) the conjugation system used to attach functional TTC to the nanoparticle utilizing PLGA-PEG-biotin functionalized biodegradable polymer, and biotinylated TTC with an avidin cross-linker, based on previously published structures [213]. Copyright 2007, Elsevier **C.** Schematic illustration of a lipid multilayer microarray. **D-F.** Uptake of DOPE-rhodamine–labeled DOTAP by NIH 3T3 cells. Reproduced with permission [214]. Copyright 2012, Elsevier.

To overcome the limitations of these methods and the adverse effects of drugs, researchers have been actively developing more effective drugs and delivery methods with lower toxicity and side effects, and drug delivery systems have received a great deal of attention, especially nanotechnology. Previous studies have demonstrated unique advantages of nano DDSs in improving delivery efficiency, reducing drug resistance and toxicity, and simplifying drug delivery, etc. Additionally, nanocarriers can encapsulate a range of drugs with diverse functions and mechanisms, thereby amplifying their synergistic therapeutic effects. Nanomedicines not only enable time- and space-specific site-specific delivery, but also provide sustainably adequate doses in a controlled-release manner (Fig. 10A–B) [127]. Nanomedicine classification encompasses drug nanoparticles, carrier-based nanomedicines, and other formulations. Various materials are employed in nanomedicine, such as liposomes, nanocrystals, nanoparticles (organic and inorganic), and polymer micelles. Among these, liposomal drugs dominate clinical applications due to their outstanding biocompatibility and sustained release characteristics. Taking advantage of their resemblance to cell membranes, liposome-based drug delivery systems efficiently traverse cellular barriers to reach target organs (Fig. 10C–F) [128].

The encapsulation of antimicrobial agents into lipid structures known as liposomes was first introduced by Bangham in 1965. Liposomes have unique properties that make them the most widely used antimicrobial drug delivery vehicles [129]. Amphotericin B, a polyene macrolide antibiotic, binds to sterols in cell membranes. Due to its limited oral absorption, intravenous administration is necessary to attain therapeutic concentrations. However, it readily binds to serum proteins and accumulates in the liver, lungs, spleen, and kidneys, causing toxicity, the use of liposomes has notably diminished the drug's adverse effects. Two liposomal formulations of amphotericin B, namely AmBisome® and Amphotec®, were introduced to the market in 1990 and 1996, respectively. These formulations were effective and well tolerated with fewer side effects than regular amphotericin B. Over time, more anti-infective liposomal products with different routes of administration have been introduced [130]. Due to the serious side effects associated with systemic antibiotic administration, including liver and kidney toxicity, disruption of beneficial gut bacteria, and the development of multi-drug resistance. To address these issues, researchers have proposed the use of bioorthogonal chemistry-based local prodrug concentration and activation strategy that improves therapeutic efficiency by locally concentrating and activating prodrugs for systemic antibiotic therapy while reducing antibiotic side effects [131]. Nano-formulations have shown effectiveness in improving drug solubility, and a large number of drug delivery carriers prepared from inorganic, polymeric and biomaterials have been reported for improving the solubility and efficacy of BCS class II and IV drugs [132].

#### 3.4.2 Extracellular vesicle-based systems

**Fig. 11:**
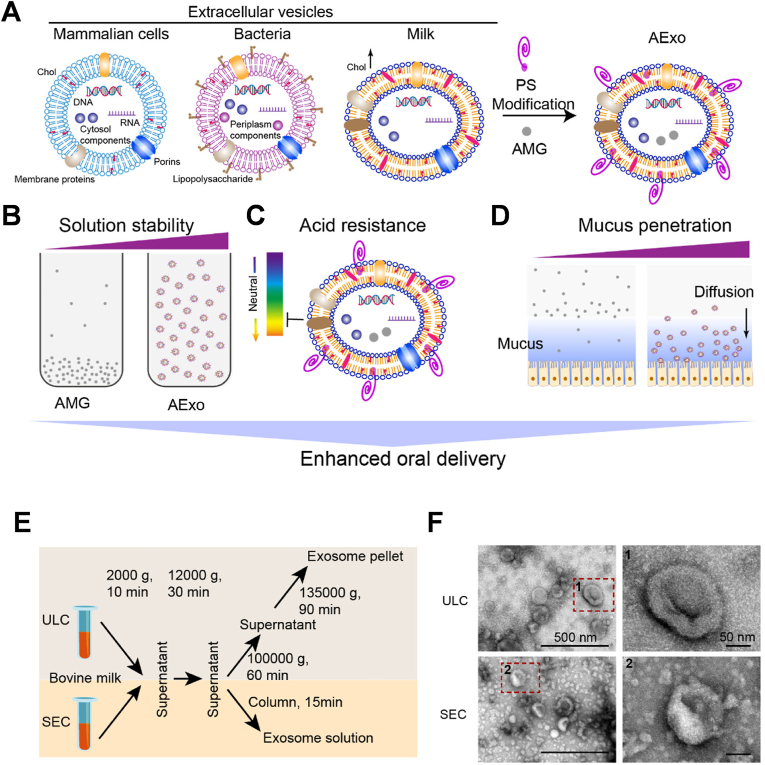
Schematic illustration of functionalized milk exosomes (mExo) for oral delivery of poorly soluble AMG to enhance the therapeutic efficacy. **A.** Schematic depiction of the structure of extracellular vesicles derived from cell lines, bacteria, and milk, respectively, for comparative analysis. mExo contains enriched Chol in total lipids to increase the membrane rigidity. mExo functionalized with PS and AMG to increase the antibiotic accumulation and enhance the antibacterial efficiency. **B−D**. AExo improves the solution stability **B**, acid resistance **C**, and mucus permeability **D** to improve the oral delivery of poorly soluble compounds. These characteristics make mExo a promising drug delivery carrier to enhance the efficacy against intestinal infections. Reproduced with permission [215]. Copyright 2022, American Chemical Society.

However, the application of these nanomaterials delivery systems is usually limited by cytotoxicity and immunogenicity. Exosomes have attracted strong attention as important mediators of intercellular communication. Exosomes, nano-sized extracellular vesicles, are released by cells in response to environmental cues or self-stimulation. They possess a lipid-membrane bilayer structure and are internalized and secreted through various mechanisms, exerting cell-specific effects. Professor Zhu's team has found that highly biocompatible milk exosomes (mExo) can substantially increase the solubility of α-invertin and significantly enhance the anti-intestinal infection efficacy, providing technical support for enhancing the solubility of BCS class II and IV drugs. The researchers successfully isolated and prepared functionalized mExo from milk, and subsequently verified in vitro that mExo could enhance the solubility of BCS class II and IV drugs and play the roles of delayed drug release and antibacterial, and found that it had the ability to significantly enhance the penetration of drugs into the mucus barrier. The mouse intestinal infection model and chicken necrotising enteritis model showed significant therapeutic effects compared with free antibacterial drugs (Fig. 11) [133]. While antibiotic liposomes have been widely used, the encapsulation and purification process is cumbersome, and there are some residues of organic reagents. Bacterial bacterial outer membrane vesicles (OMVs) have excellent biocompatibility and like-for-like targeting, and have great potential for application in drug loading. OMVs are small vesicles released from the outer membrane of the bacterial cell, which contain membrane proteins and biologically active substances. Researchers have harnessed OMVs as a drug delivery carriers, loading antibiotics into them by mimicking the mechanisms bacteria employ to counteract drugs. This approach enhances the stability and targeting of drugs, leading to the development of novel nano-antibiotics that tackle bacterial drug resistance. This novel drug delivery strategy opens up new avenues for antibiotic research and therapy.

As DDSs continue to evolve, significant advances have been made in the field of antibiotic delivery, especially with the rapid development of nanomedicine and the growing understanding of disease mechanisms, these technologies are improving therapeutic efficacy while minimizing the development of drug resistance and reducing adverse drug reactions, enabling a more precise therapeutic approach to improve the quality of life of patients. These advances are not only revolutionize traditional drug delivery methods but also provide new strategies and solutions for the treatment of chronic diseases and acute infections.

### 3.5 Cancer pain and drug delivery systems

Pain is a complex sensory and emotional experience that has a significant impact on a patient's quality of life. Cancer pain management endeavors to alleviate pain and enhance the patient's quality of life through diverse methodologies. Commonly used pain treatments include oral medications, subcutaneous injections, and topical medications. Oral medication is the mainstay of this, and includes commonly used analgesic medications such as opioids, NSAIDs, and adjunctive medications such as antidepressants and antiepileptic drugs. However, these treatments have limitations, such as inaccurate dosage control, digestive side effects, and short-term effectiveness, and the development of drug delivery technologies has opened up new possibilities for pain management, aiming to improve efficacy and reduce side effects by enabling precise delivery of medications.

#### 3.5.1 Transdermal delivery system

The Transdermal Delivery System (TDDS) is a non-invasive method of administering medication through the skin into the bloodstream. It can avoid the first-pass effect of the liver and gastrointestinal tract damage associated with oral administration, while reducing fluctuations in blood medication levels, decreasing adverse reactions, and enhancing the convenience and compliance of patient medication use. One of the earliest DDSs used in pain management was the transdermal delivery system, also known as patches. This system releases the drug by applying it to the surface of the skin, enabling gradual absorption of the drug to provide sustained analgesia. The microneedle platform is a novel transdermal drug delivery system, which can deliver drugs directly into the superficial tissues beneath the skin surface through the sub-millimetre microneedle design, thus achieving effective and painless drug delivery and local therapeutic effects, avoiding the circulatory and metabolic processes of drugs in the body required for oral drug delivery, and the local delivery reduces the side-effects of the drugs in the body and improves the therapeutic efficacy [134]. This treatment is commonly used in the treatment of traumatic wounds, through the slow and sustained release of drugs to achieve a long-lasting analgesic effect. The micro-needle platform efficiently clears wound surface debris and bacteria, facilitating drug delivery and treatment. Direct delivery of the drug to the wound area promotes repair and healing, reducing pain and inflammation.

#### 3.5.2 Nanodrug delivery systems

Moreover, the emergence of nanomedicine presents new avenues for analgesic application. By manipulating the structure, size, and surface properties of nanomaterials, researchers can optimize the pharmacokinetics and biodistribution of drugs, enhancing drug delivery and therapeutic outcomes. Encapsulating drugs within nanoscale carriers enables precise targeting and controlled release, elevating local drug concentrations, diminishing systemic side effects, and extending sustained drug release [135]. Epidural administration is commonly used to treat pain, with ibuprofen having potential applications. Due to its short duration of action, researchers are developing a long-acting liposomal gel injection of ibuprofen to improve pain management. The gel, prepared using Poroxam 407 and a solution of high-pressure homogenisation-treated ibuprofen liposomes, significantly prolongs drug release, controls drug penetration in the dura mater, and has good gel stability. This novel liposome gel shows potential as a controlled-release DDS [136]. Enkephalin, an endogenous neuropeptide that activates MOPr (mu opioid peptide receptor) and DOPr (delta opioid peptide receptor), is a potential candidate for novel analgesics. Feng et al. have investigated the development of a novel analgesic nanomedicine designed to bypass the blood-brain barrier and produce analgesia using morphine. Nanoparticles are used as carriers to deliver morphine, allowing the drug to cross the blood-brain barrier and release morphine in the central nervous system. The nanomedicine had a faster onset of action and a longer analgesic effect than conventional morphine while reducing side effects such as respiratory depression and drug resistance [137]. In 2020, Nestor N Jimenez-Vargas et al. innovated a nanoparticle delivery approach targeting selective activation of DOPr within injury receptor endosomes, effectively inducing prolonged suppression of neuronal excitability for inflammatory pain management [138]. In the same year, Prof. Bu and Liu innovatively proposed “Tumor-Nerve Communication Blockade” from the perspective of “Tumor Neurology” for the first time. With the help of designed and constructed functional nanomaterials, it can cut off the signal communication between metastatic bone tumors and their peripheral nerves to achieve efficient treatment of metastatic bone cancer pain. Given the limited efficacy of inflammation-based pain therapies for cancer-related pain, this study aimed to fabricate a multifunctional nanocomposite capable of disrupting bidirectional signaling between bone tumors and peripheral nerves. This approach introduces a promising strategy for inhibiting tumor progression while concurrently addressing metastatic bone cancer pain. Upon targeting and infiltration into the bone tumor site, the LDH promptly reacts with surplus hydrogen ions (H), thereby diminishing the nociceptive stimulation of the nerves adjacent to the tumor, consequently yielding a swift analgesic response. Consequently, this strategy not only efficaciously alleviates cancer-associated pain but also facilitates bone repair, elevating cancer pain thresholds by nearly 30 % compared to the control group, thereby underscoring the substantial potential of this innovative nanomedicine approach to cancer pain management [139].

#### 3.5.3 Long-acting DDSs

**Fig. 12:**
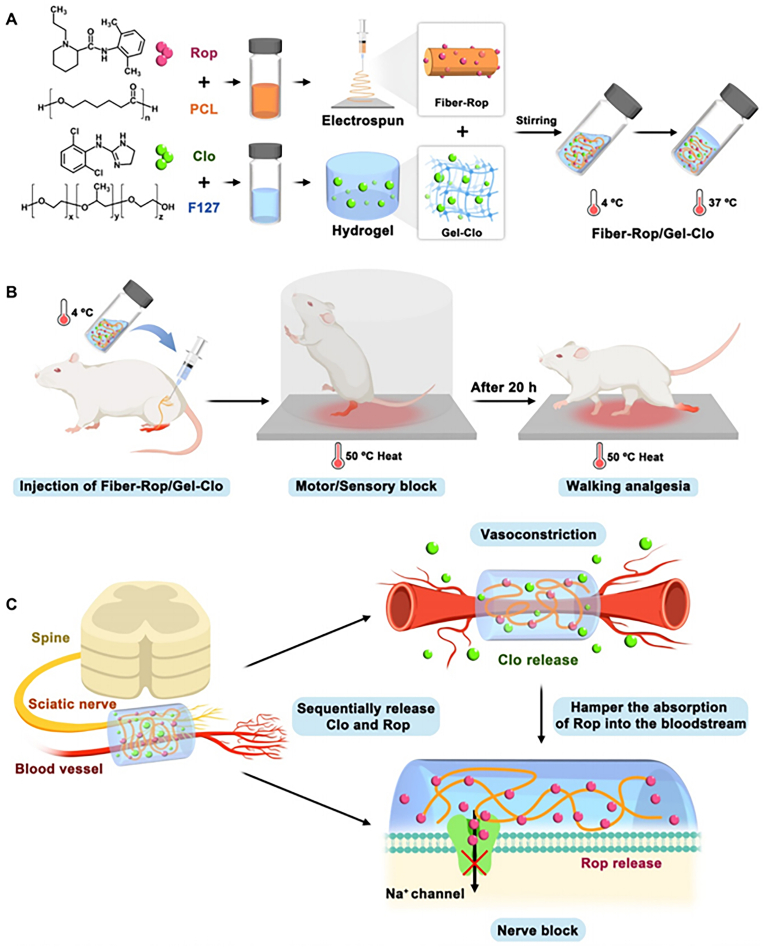
Schematic illustration of fabrication and sciatic nerve blockade effect of the Fiber-Rop/Gel-Clo composite. **A.** The Fiber-Rop/Gel-Clo composite was fabricated by combining Rop-loaded electrospun PCL nanofiber and Clo-loaded F127 hydrogel. **B.** The rat sciatic nerve blockade effect of the Fiber-Rop/Gel-Clo composite. **C.** The mechanism of long-acting and walking regional analgesia in vivo. Reproduced with permission [216]. Copyright 2022, Theranostics.

Long-acting Drug Delivery Systems (DDSs) are dedicated to prolonging the therapeutic effect of medications and reducing the frequency of administration. They maintain the necessary therapeutic concentrations in the bloodstream or tissues by modulating the drug's clearance rate, typically adjusting pharmacokinetic parameters such as the time to reach maximum concentration (Tmax) and the maximum concentration (Cmax). Ultimately, these systems achieve an extended duration of drug action and decrease the frequency of dosing. By providing a sustained release of medication, they not only improve patient compliance but also have the potential to enhance therapeutic outcomes. Long-acting DDSs have become a significant focus of current research in the field of pain management. Through the use of extended-release technology and implantable devices, sustained release of drugs can be achieved, reducing the frequency of frequent medication for patients. This is not only convenient for patients, but also provides a more stable therapeutic effect. Transdermal patches and implantable pumps are widely used in pain management. For instance, Zhang and colleagues devised an injectable composite of hydrogel and microspheres (GEL/MS), simultaneously encapsulating bupivacaine (BUP) and dexamethasone (DEX). The researchers did this by designing a complex consisting of two parts, a hydrogel and microspheres, with the hydrogel containing dexamethasone and the microspheres containing bupivacaine. The complex sequentially releases dexamethasone, an α2-adrenergic receptor agonist with analgesic and sedative properties, and bupivacaine, a local anaesthetic providing local anaesthesia for prolonged and synergistic analgesic effects. The results showed that the complex was able to sustain the release of dexamethasone and bupivacaine, and that the release of dexamethasone inhibited the inflammatory response and prolonged the analgesic effect of bupivacaine. The results of this study suggest that this long-acting hydrogel/microsphere complex has potential for use in clinical analgesic therapy and opening up new possibilities for treatment in the field of pain management [140]. Chen et al. designed an injectable electrostatically spun fibre-hydrogel composite capable of sequentially releasing clonidine and ropivacaine. The functions of clonidine and ropivacaine were similar to those of DEX and BUP, respectively. This composite, composed of clonidine-containing nanofibers and a hydrogel containing ropivacaine, achieved prolonged regional analgesia in a mouse model. This development opens new possibilities for regional analgesic therapy (Fig. 12) [141]. In summary, pain management and drug delivery are a constantly evolving and innovative field of research. By harnessing nanotechnology, long-acting delivery systems and other novel delivery technologies, we are continually striving to improve the efficacy and safety of medications, providing more effective treatment options for individuals experiencing pain.

### 3.6 Tumor immunotherapy and drug delivery systems

Despite the revolutionary advances in the field of tumor therapy brought by immunotherapy, the rate of clinical patients benefiting from the treatment is low, with a good therapeutic effect on only 15 % of patients. This can be attributed to the complex tumor immune microenvironment and the immune escape mechanism of tumors [142]. In addition, systemic immunotherapy lacks precise targeting of lesions and can cause damage to normal tissues, thus causing a series of immune-related adverse reactions that not only negatively impact the effectiveness of the treatment, but in serious cases, may even endanger the patient's life.

**Fig. 13:**
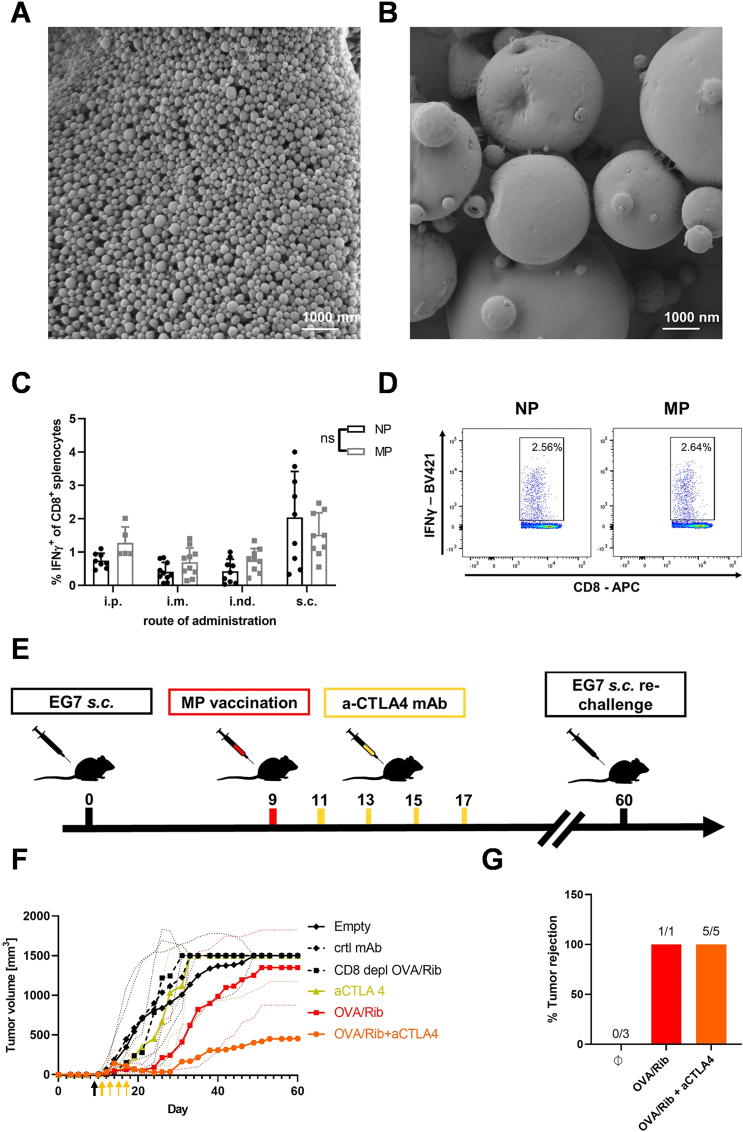
Nano- or micron-sized PLGA particles containing ovalbumin (OVA) and Riboxxim are potent vaccine delivery systems for vaccination. **A, B.** Size distribution and morphology of nanoparticles **A** and microparticles **B** containing encapsulated OVA and Riboxxim were analyzed by scanning electron microscopy (SEM). Scale bars, 1000 nm. SEM images were acquired from three different particle batches with similar results. **C**. C57BL/6J mice were immunized with nanoparticles (NP, black dots) or microparticles (MP, gray squares) charged with OVA protein (250 μg/mouse) and Riboxxim (2.5 μg/mouse) via the intraperitoneal (i.p., n = 8 for NP, n = 5 for MP), intramuscular (i.m., n = 10 for NP, n = 10 for MP), intranodal (i.nd., n = 9 for NP, n = 9 for MP), or subcutaneous (s.c., n = 9 for NP, n = 9 for MP) route demonstrating the latter as most efficient. Six days post immunization, an intracellular cytokine staining for IFNγ+ of CD8^+^ splenocytes was performed and analyzed via flow cytometry. **D.** A representative dot plot showing the frequency of IFNγ+CD8^+^ splenocytes for indicated treatment groups. **E**. PLGA-microparticles (MP) and immune checkpoint blockade combination therapy. **F** Tumor volume **G** Tumor rejection. Reproduced with permission [217]. Copyright 2021, Nature Publishing Group.

To address challenges associated with tumor immunotherapy, researchers have begun to combine cancer immunotherapy with DDSs, which has been shown to improve drug efficacy by precisely targeting drug delivery, mitigating drug side effects, and improving pharmacokinetics. The application of nanotechnology is particularly prominent, through the design of smart nanocarriers, ICIs can be precisely delivered to specific sites to implement safer and more controllable tumor immunotherapy, which greatly enhances the continuity and safety of treatment [143]. In order to enhance the efficacy of immunotherapy, Marcus Groettrup et al. developed a vaccine based on PLGA particles carrying Riboxxim to activate the TLR3 and RIG-I pathways, taking advantage of its ability to gradually release the drug. This strategy of combining such a vaccine with an immune checkpoint inhibitor aims to address the problem of drug resistance that is common when treatment is given alone, as well as to enhance therapeutic efficacy (Fig. 13) [144].

#### 3.6.1 ICIs

**Fig. 14:**
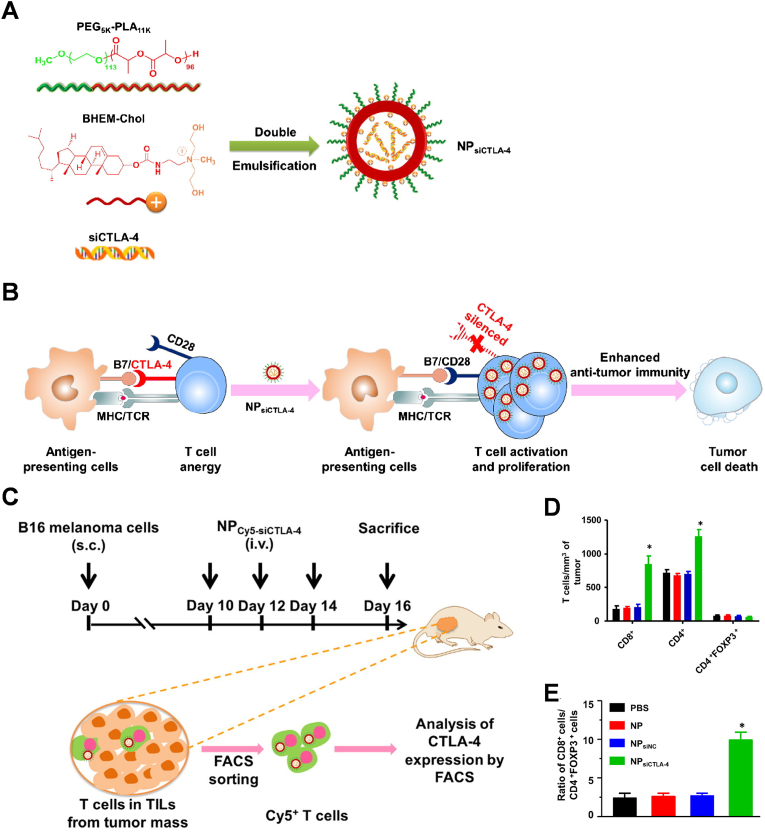
**A.** Preparation of siCTLA-4-encapsulated nanoparticles (NPsiCTLA-4) with poly (ethylene glycol)-block-poly (D, L-lactide) and a cationic lipid BHEM-Chol by double emulsification. **B.** Enhancing T cell-mediated immune responses by blocking CTLA-4 using NPsiCTLA-4. CTLA-4 plays a strong inhibitory role in T cell activation and proliferation, which significantly curbs T cell-mediated tumor rejection. NPsiCTLA-4-mediated CTLA-4 knockdown enhanced the activation and proliferation of T cells, which inhibited the overall growth of tumors. **C.** the expression of CTLA-4 on Cy5 positive CD4^+^ T cells. **D, E.** The Cy5-positvie population of CD4^+^ and CD8^+^ T cells was analyzed in five individual experiments. Reproduced with permission [218]. Copyright 2016, Elsevier.

ICIs, such as CTLA-4 and PD-1/PD-L1 inhibitors, have emerged as the most extensively studied and utilized immunotherapies. Numerous ongoing trials are exploring checkpoint inhibitors in conjunction with chemotherapy or targeted agents. Nevertheless, the systemic administration of checkpoint inhibitors may induce severe multi-organ side effects, while challenges such as limited tumor penetration and lower tumor cell uptake can compromise the efficacy of oncology treatment [145]. The use of polymeric pLHMGA particles as a sustained and local release system for anti-CD40 and anti-CTLA-4 antibodies in cancer immunotherapy was found to be effective in sustaining the release of antibodies into the tumor microenvironment in experiments on a murine model of colorectal cancer, thereby sustaining their anti-tumor effect and reducing systemic side effects [104]. Another study focused on colorectal cancer liver metastases, in which researchers developed an alternative method of delivering immunotherapeutic agents by delivering pDNAs engineered with PD-L1 traps and/or CXCL12 traps to the nucleus of liver cells via calcium lipid phosphate nanoparticles. This approach significantly increased the concentration of immunotherapeutic agents in local tissues, allowing the therapy to reduce the accumulation of immunosuppressive cells and show superior efficacy in treating liver metastases with high efficacy and reduced off-target toxicity [146]. T-cell exhaustion is an important limiting factor in the immune response process, especially in the tumor microenvironment. Overexpression of immune checkpoint molecules (e.g. PD-1) usually leads to suppression of T-cell function and fatigue, hampering an effective anti-tumor immune response. Professor Gu's team research focuses on a novel approach to cancer immunotherapy using a microneedle-based system for transdermal delivery of checkpoint inhibitors, the researchers have developed a microneedle system capable of delivering therapeutic agents directly into the tumor microenvironment. The system uses a combination of anti-PD1 antibodies and 1-methyl-dl-tryptophan (1-MT, an inhibitor of the immunosuppressive enzyme IDO) encapsulated in a nanocarrier made from 1-MT-modified hyaluronic acid. The approach aims to enhance the retention and slow release of these drugs at the tumor site, thereby maximising therapeutic efficacy while minimizing systemic exposure. The study suggests a potential new therapeutic strategy that may be more effective and less harmful than current systemic treatments [147]. A primary objective of tumor immunotherapy involves the prolonged activation and proliferation of tumor-specific T cells, specifically cytotoxic T lymphocytes (CTLs) that infiltrate the tumor. Li et al. fabricated nanoparticles for delivering cytotoxic T lymphocyte-associated molecule-4 (CTLA-4)-siRNA (NPsiCTLA-4) in a murine melanoma investigation. The findings indicated that this nanoparticle delivery system successfully transported CTLA-4-siRNA to CD4(+) and CD8(+) T-cell subsets at the tumor site, markedly enhancing the proportion of anti-tumor CD8(+) T-cells and diminishing the ratio of suppressor T-regulatory cells (Tregs) among tumor-infiltrating lymphocytes (TILs). This led to augmented activation of tumor-infiltrating T-cells and anti-tumor immune responses. The results demonstrate the efficacy of this siRNA delivery system in infiltrating T-cells both in vitro and in vivo, highlighting its considerable potential for nanoparticle-based cancer immunotherapy in melanoma treatment (Fig. 14) [148].

**Fig. 15:**
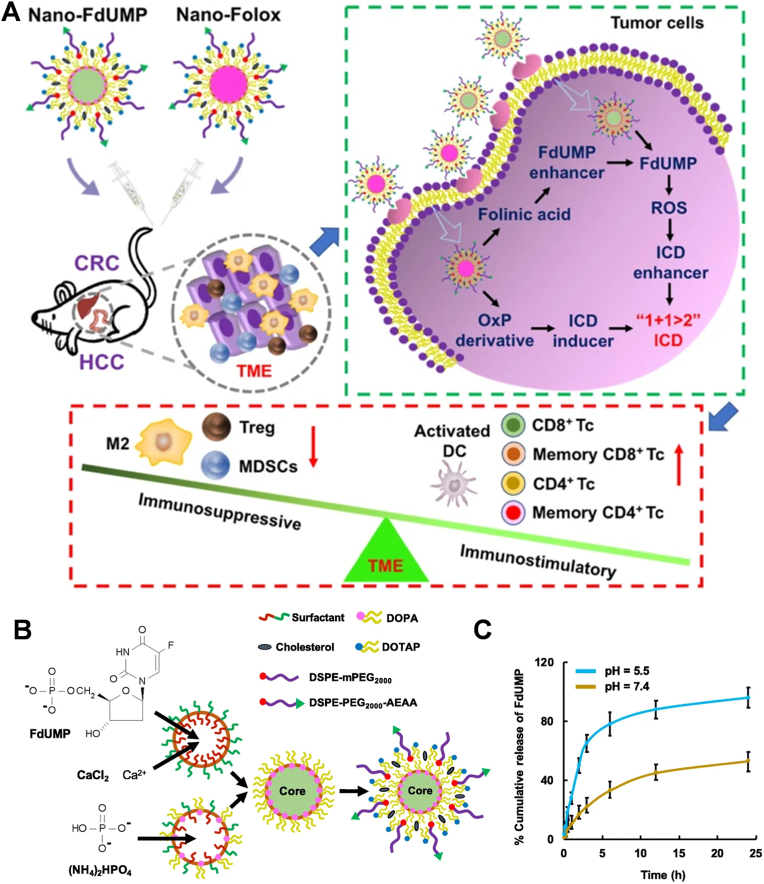
**A.** Graphical abstract of synergistic immunotherapy. **B.** Nano-FdUMP developed in microemulsions using nanoprecipitation technique. **C.** The in vitro release of fluorine drug from nanoprecipitates within Nano-FdUMP in pH = 5.5 and 7.4(n = 4). Reproduced with permission [219]. Copyright 2021, Springer Nature.

The combination of chemotherapy and immunotherapy has shown promising results in the treatment of colorectal cancer (CRC) and hepatocellular carcinoma (HCC). The FOLFOX regimen, consisting of folinic acid (FnA), fluorouracil (5-Fu), and oxaliplatin (OxP), has been the standard of care for these cancers. In a study, nanoprecipitation technology was used to develop a nano-agent (called Nano-Folox) for colorectal cancer and HCC treatment containing OxP derivatives and FnA, which act directly on the tumor cells, triggering intracellular reactive oxygen species (ROS) and immunogenic cell death, a form of immunogenic cell death. This immunogenic cell death not only leads to the elimination of tumor cells, but also activates the immune system and enhances the anti-tumor immune response, which plays an important role in both synergistic chemotherapy and immunotherapy, and the results of the study demonstrating significant anti-tumor effects in both in vitro and in vivo models, thus effectively eliminating CRC and HCC (Fig. 15) [149].

#### 3.6.2 Cancer vaccines

**Fig. 16:**
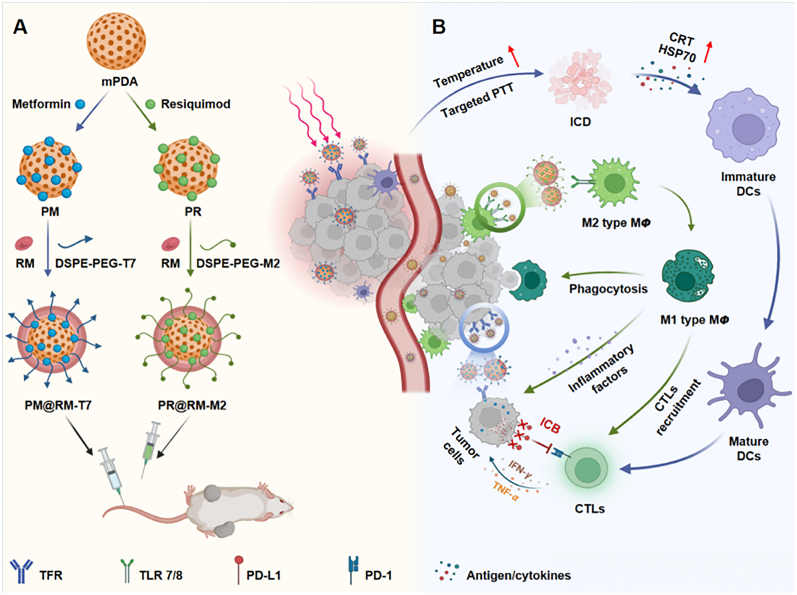
**A**. the construction of the biomimetic “Gemini nanoimmunoregulators” and **B**. the synergistic photoimmunotherapy via spatiotemporal targeted PTT, phenotype reversion of TAMs, and PD-L1 inhibition. Reproduced with permission [220]. Copyright 2024, Elsevier.

Cancer vaccines are an active immunisation approach that harnesses the power of the immune system to induce tumor-specific T cells against cancer in patients, and could be developed as either prophylactic tools to prevent future recurrences of cancer or as therapeutic treatments to boost the immune system's ability to eliminate tumors. However, developing effective cancer vaccines comes with additional challenges, such as precise binding of humoral and cellular immunity, orderly activation of immune components, and immune tolerance or hyporesponsiveness [150]. Nanotechnology approaches are uniquely positioned to address the challenges of vaccination and unlock the potential for additional applications in cancer therapy, with vaccine nanotechnology having multifunctional properties based on its customised structures, modular compositions and controllable length scales. In 2016, researchers used Papaya Mosaic Virus (PapMV)-derived nanoparticles to enhance the potential of cancer immunotherapy. The team found that by exploiting the nanoparticle nature of PMV, a novel cancer treatment strategy could be devised, an approach that fights cancer cells by activating the immune system. These PMV nanoparticles are biocompatible and stable enough to be effectively recognised by the immune system, and by loading specific tumor antigens or immune-stimulating molecules onto PMV nanoparticles, the immune system's recognition and attack of tumor cells can be enhanced. This approach not only enhances the immunogenicity of tumor cells, but also activates specific immune cells, such as T cells and dendritic cells, to trigger an immune response against the tumor. It holds promise as a novel strategy for cancer prevention and treatment [151]. Kuai et al. have devised a novel personalized cancer immunotherapy strategy employing high-density lipoprotein (HDL)-mimicking nanodiscs, proficiently co-delivering antigenic peptides and adjuvants to lymph nodes. These nanodiscs persistently present antigens on dendritic cells, fostering the production of CTLs targeting tumor antigens, thus combating tumor cells. Moreover, it was observed that when coupled with anti-PD-1 and anti-CTLA-4 therapies, this nanodisc vaccine effectively eradicated established MC-38 and B16F10 tumors [152]. Professor Ding's team utilized DNA origami technology to construct a tumor vaccine system with precisely controllable size and shape. By precisely assembling two molecular adjuvants and an antigenic peptide within the tubular DNA nanostructures, they formed a well-defined DNA nanodevice vaccine. This vaccine leverages antigen-specific immune responses for tumor immunotherapy. It has shown good antitumor efficacy in mouse models of melanoma and colorectal tumors and has demonstrated long-term immune memory effects, effectively inhibiting tumor recurrence and metastasis [153]. Recently, Professor Wu's team has developed a novel bionic “Gemini nano-immunomodulators”, PM@RM-T7 and PR@RM-M2, which are designed to achieve potent optical-immune combination therapy by spatiotemporal targeting of tumor cells, PD-L1 checkpoints, and M2-type TAMs to inhibit tumor growth and metastasis effectively.PM@RM-T7 (the first type of nanomodulators) is designed to trigger amplified immunogenic cell death (ICD) effects and effectively activate anti-cancer immune responses by targeting photothermal therapy (PTT). Meanwhile, PD-L1 on remaining cancer cells gets degraded by burst Met catabolism to prevent immune escape. PR@RM-M2 (the second nanoregulator), on the other hand, specifically recognised TAMs and reprogrammed their phenotype from M2-type to M1-type, thereby breaking the immunosuppressive microenvironment and further enhancing the function of cytotoxic T-lymphocytes. The combined effect of the two nanoregulators not only markedly suppressed the growth of both primary and distant tumors but also hindered tumor metastasis The combined effect of these nanoregulators suppresses tumor growth, inhibits metastasis, and offers a new approach for effective cancer immunotherapy targeting metastatic tumors (Fig. 16) [154].

#### 3.6.3 Other types of immunotherapy

**Fig. 17:**
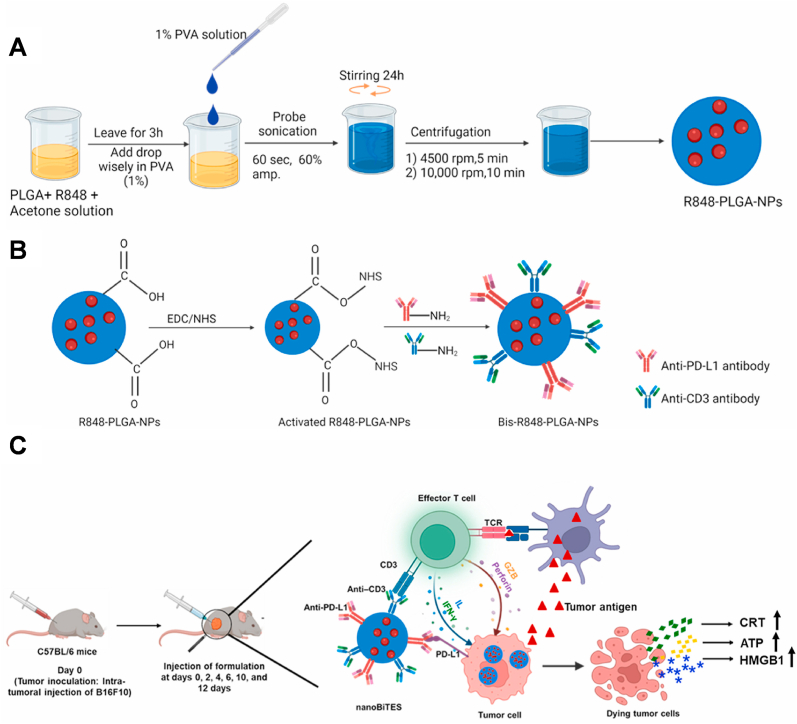
**A.** Synthesis of R848-PLGA-NPs, **B.** Synthesis of bis-R848-PLGA-NPs **C.** Schematic illustration mechanism of bis-R848-PLGA-NPs to activate the antitumor immune response in B16F10 xenograft mice model. In vivo analysis of GZB and perforin, expression in CD8^+^ T cells of mice tumor after the administration of free PLGA-NPs, R848-PLGA-NPs, anti-CD3-R848-PLGA-NPs, anti-PD-L1-R848-PLGA-NPs, and bis-R848-PLGA-NPs. Reproduced with permission [221]. Copyright 2022, Elsevier.

In recent years, cell-based DDSs have been shown to outperform conventional delivery systems and has been rapidly evolving [155]. Various types of cells can be used for cell delivery, including red blood cells, platelets, stem cells, and immune cells. In the context of tumor immunotherapy, classical cell therapies such as chimeric antigen receptor T (CAR-T) cells, chimeric antigen receptor natural killer (CAR-NK) cells, and T-cell receptor-engineered T (TCR-T) cells operate by harvesting host immune cells, subsequently modifying them in vitro, and reintroducing them into the body to target and eliminate tumor cells [156]. However, CAR-T cell therapy also has many limitations, for example, it can induce severe toxic side effects such as cytokine release syndrome (CRS) and neurological toxicity. Additionally, it may inadvertently harm normal tissue cells while attacking tumor cells, resulting in adverse reactions. Some tumor cells may even escape CAR-T cell attack by changing surface antigens or other mechanisms, leading to treatment failure or tumor recurrence. In recent years, platforms for the development of nanomedicine carriers have been reported to couple CAR-T cell surface-modified nanoparticles or nanobodies to BiTE for probe-like targeting, with good results in preclinical experiments (Fig. 17) [157]. Oncolytic virus therapy (OVT) has attracted much attention as an emerging tumor immunotherapy. Oncolytic viruses (OVs) are live, attenuated viruses capable of specifically infecting and destroying tumor cells with little effect on normal cells. By selectively introducing therapeutic transgenes into the tumor microenvironment (TME), OVs are able to enhance anti-tumor effects and immune responses [158]. In a phase II clinical trial investigating the preoperative administration of T-VEC combined with neoadjuvant chemotherapy, 45.9 % of patients attained complete remission, with 89 % of patients remaining recurrence-free two years post-treatment. Moreover, the capacity of oncolytic viruses to selectively infect tumor cells and provoke a systemic anti-tumor immune response has been recognised as a promising immune adjuvant to augment the effectiveness of other tumor immunotherapies, including immune checkpoint inhibitors [159]. The integration of immunotherapy and DDSs holds promise for enhancing drug specificity and safety. Therefore, the development of novel nano-drug delivery system that merges the benefits of immunotherapy and DDSs significantly enhances drug targeting precision, offering renewed hope for immunotherapy in treating solid tumors.

## 4 Challenges of DDSs in the treatment of cancer complications

### 4.1 Challenges and limitations

**Fig. 18:**
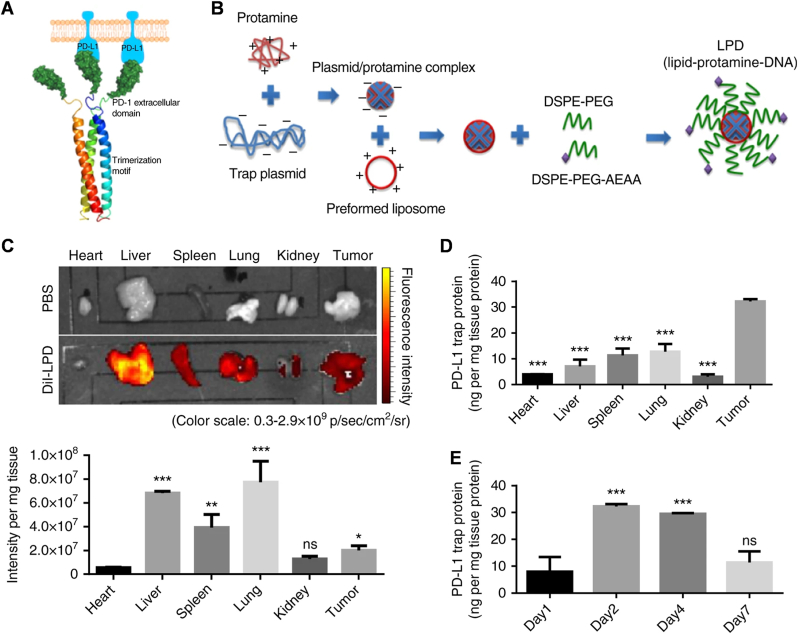
Design of locally and transiently expressed PD-L1 trap. **A.** Scheme showing the tribody interaction of PD-L1 trap protein. **B.** Preparation scheme of PD-L1 trap plasmid loaded LPD. **C.** Images and quantitative results of the DiI-loaded LPD in major organs and the CT26-FL3 tumor at 24 h after injection (n = 3). **D.** PD_x0002_L1 trap protein expression in major organs and the CT26-FL3 tumor at 48 h after injection. The PD-L1 trap expression was measured using ELISA by targeting the His (6 × )-tag engineered at the C-terminus of the PD-L1 trap (n = 3). **E.** PD-L1 trap protein expression in tumors on days 1, 2, 4 and 7 after injection. Significant differences in c, d, and e were assessed using *t*-test. Results are presented as mean (SD). ns, not significant. *P < 0.05, **P < 0.01, ***P < 0.001. Reproduced with permission [222]. Copyright 2018, Nature Publishing Group.

As novel drug delivery systems continue to be developed and applied, significant breakthroughs and progress have been achieved in the field of tumor treatment. These advancements hold the potential to substantially improve therapeutic outcomes, improve patient quality of life, and subsequently extend patient survival. However, drug delivery in the treatment of tumor complications also faces several challenges. 1) Complexity of the TME: TME constitutes the internal environment produced by and supporting tumor cells. It comprises a diverse assembly of components, including various immune cells, cancer-associated fibroblasts (CAFs), endothelial cells, and inflammatory/immune cells, as well as non-cellular elements like the extracellular matrix (ECM) and cytokines [160]. The composition of these components can differ based on tissue type and may evolve in tandem with tumor progression. Furthermore, the cellular composition and functional status of the TME can vary extensively depending on the originating organ of the tumor, the intrinsic properties of the cancer cells, the stage of the tumor, and patient-specific characteristics. These factors may directly or indirectly impact the distribution and therapeutic efficacy of drugs within tumor tissues. 2) Drug Metabolism and Resistance: The effectiveness of drug delivery is often compromised by various pharmacokinetic challenges, including absorption, distribution, metabolism, excretion, and drug interactions. These factors can prevent drugs from being efficiently delivered to targeted lesions within the body, resulting in subtherapeutic drug concentrations at the intended sites and consequently affecting treatment outcomes. These constraints significantly impact the pharmacological efficacy and safety of drugs and must be meticulously considered and addressed during the design and optimization of drug delivery systems. Furthermore, tumor cells frequently develop diverse mechanisms of resistance to chemotherapy agents and other therapeutic interventions. This resistance can be an inherent characteristic of the tumor or may evolve during treatment. Tumor cells can develop resistance to cytotoxic drugs through several pathways, with key mechanisms involving drug metabolism and inactivation, crosstalk between kinase signaling pathways, alterations in cell cycle and checkpoint regulation, evasion of the immune system, DNA damage and repair processes, and epigenetic modifications [161]. Drug resistance can result in decreased sensitivity of tumor cells to therapeutic agents, leading to the ineffectiveness of previously successful treatments and potentially causing tumor recurrence or progression. Consequently, there is a critical need to develop new drug delivery systems that improve the stability and selectivity of drugs, extending their effective duration to enhance therapeutic outcomes. 3) Design and Fabrication of Drug Delivery Systems: The design and fabrication of DDSs face several challenges, including the selection of appropriate drug carriers, ensuring precise drug targeting, and the flexible control of drug release. To achieve enhanced therapeutic efficacy, various delivery technologies have been developed, such as combination therapy and coupling technologies. Integrating chemotherapeutic agents with PD-L1 inducers has been shown to enhance immunogenicity and immune response against tumors, offering potential for effective cancer immunotherapy with reduced adverse reactions (Fig. 18) [162]. The application of conjugation technologies has facilitated the precise delivery of chemotherapeutic agents, substantially mitigating their systemic toxicity. Antibody-Drug Conjugates (ADCs) and Peptide-Drug Conjugates (PDCs) currently stand as the most extensively researched variants of these conjugation-based drug delivery systems, functioning as carrier-independent molecular delivery platforms [163]. However, each coupling technology comes with its own set of limitations. ADCs, for instance, are prone to off-target risks [164]. While PDCs mitigate some of the drawbacks associated with ADCs, the short biological half-life of peptides limits the distribution and targeting duration of PDCs within the body, thereby restricting the efficiency of effective payload delivery to tumor cells [165]. The advancement of drug delivery research not only confronts the aforementioned challenges but also grapples with issues related to drug stability during the delivery process. Factors such as enzymatic degradation, photosensitivity, and chemical reactions can lead to the inactivation or degradation of drugs. Biocompatibility of the delivery systems is also crucial to ensure safety and tolerance. Moreover, optimizing drug delivery efficiency and drug permeability are areas that require further optimization. Cost optimization of delivery materials is also a challenge for the clinical translation of these technologies. Therefore, for various types of tumor complications, it is imperative to design DDSs with specific targeting capabilities that ensure precise delivery of drugs to tumor tissues and the release of adequate drug concentrations to achieve therapeutic effects.

### 4.2 Future prospects for DDSs

Despite the challenges in DDSs, advancements in nanotechnology and bioengineering are driving their evolution towards greater precision and personalization. These advancements hold promise for addressing tumor-related complications, especially in the field of immunotherapy. However, there is limited research on complications related to other types of tumors. Chemically synthesized materials, particularly lipid nanoparticles, are widely studied in drug delivery systems (Table 3). In future studies, it is crucial to not only focus on the treatment of the primary tumor lesion, but also pay more attention to the treatment of tumor complications. Continuous development of DDSs is expected to provide more effective solutions to these clinical challenges. Future drug delivery studies should focus more on the improvement of the following aspects: 1) Enhanced Targeting: Future drug delivery systems will increasingly focus on targeting accuracy, delivering drugs to specific tissues or cells, improving drug bioavailability within tumor tissues, and minimizing damage to healthy tissue.2) Intelligent Control: Future DDSs will incorporate intelligent control features that adjust the drug release rate in real time based on changes in the internal body environment. This capability will ensure optimal drug effectiveness over necessary durations, improve therapeutic outcomes, and minimize adverse effects. 3) Multifunctionality: Future DDSs are anticipated to be multifunctional, capable of delivering multiple drugs simultaneously, diagnosing disease states, and monitoring treatment efficacy. This integration is aimed at facilitating both therapy and continuous patient monitoring. 4) Personalized Treatment: Advances in genomics and bioinformatics are providing new paradigms for drug delivery. Customized drug delivery strategies can be formulated based on genetic testing and other individual patient data, enhancing therapeutic effectiveness while reducing adverse reactions. 5) Innovative Delivery Carriers or Technologies: There is a continuous need to explore and develop new carriers with improved drug loading capacity, tissue compatibility, and biodegradability to effectively target the appropriate cells. 6) Interdisciplinary collaboration: Future research needs to enhance the integration of multidisciplinary research to jointly advance the field of DDSs, such as joint research with materials science, biology, pharmacy, engineering, and computer science. Future research will focus on enhancing drug delivery technologies and devising innovative solutions to overcome existing challenges. Consequently, designing DDSs that are structurally simple, readily scalable for industrial production, biocompatible, highly targeted, and efficient, all while ensuring biosafety, represents a significant ongoing challenge in cancer treatment research.

**Table 3:** Nearly a decade in the literature to describe different drug delivery research of tumor associated complications.

Classification	Different drug delivery systems		Drug	Publication Date	Reference
Cancer-associated Thrombosis	Nanoparticles	Biomimetic MnOx/Ag2S nanoflower platform	Hirudin	2022 Nov	[120]
Cancer-associated Thrombosis	Nanoparticles	Platelet-membrane-coated biomimetic nanocarrier	Bortezomib	2016 Nov	[166]
Cancer-associated Thrombosis	Nanoparticles	Fibrinolytic nanocages	Doxorubicin	2021 Oct	[167]
Cancer-associated Thrombosis	Nanoparticles	Nano polymer conjugate	Doxorubicin and nattokinase	2020 May	[168]
Cancer-associated Thrombosis	Blood platelets	Platelet membrane-coated nanodrug	Doxorubicin	2023 Apr	[169]
Cancer-associated Thrombosis	Bacteria	Attenuated Escherichia coli	Cytolysin A protein	2022 May	[170]
Malignant serous effusion	Nanoparticles	Liposomes	/	2019 May	[171]
Malignant serous effusion	Nanoparticles	Liposomal Nanoparticle	Cyclic Dinucleotide	2022 Feb	[125]
Malignant serous effusion	Microparticles of cells	Tumor cell-derived microparticles	Methotrexate	2019 Jan	[121]
Malignant serous effusion	Microparticles of cells	Tumor cell-derived microparticles	Methotrexate	2020 Sep	[123]
Tumor-associated infections	Nanoparticles	Mesoporous Silica Nanoparticles	Ofloxacin, Doxorubicin and Epirubicin	2022 Jan	[172]
Tumor-associated infections	Nanoparticles	Dual-antigen-displaying nanovaccines	/	2024 Apr	[173]
Tumor-associated infections	Nanoparticles	Hyaluronic Acid and Ciprofloxacin-based self-assembling prodrug	Ciprofloxacin	2022 Dec	[173]
Tumor-associated infections	Nanoparticles	Mesoporous Silica Nanoparticles	Doxorubicin and Antimicrobial Peptide HHC36	2023 Mar	[174]
Tumor-associated infections	Other delivery systems	Intrathecal Drug Delivery System	/	2020 Jul	[175]
Cancer pain	Nanoparticles	Phototrigger able liposomal device	Tetrodotoxin	2015 Dec	[176]
Cancer pain	Nanoparticles	Gold nanorods attached to low temperature sensitive liposomes	Tetrodotoxin and dexmedetom idine	2017 Feb	[177]
Cancer pain	Nanoparticles	8D3-PLGA nanoparticles	Loperami de	2015 Aug	[178]
Cancer pain	Nanoparticles	Squalene	Leu-enkephalin	2019 Feb	[137]
Cancer pain	Nanoparticles	Mg/Al layered-double-hydroxide nanoshells	AZ-23+alendronate	2022 Apr	[139]
Cancer pain	Nanoparticles	Mesoporous silica nanoparticles and liposomes	DADLE	2020 Jun	[138]
Cancer pain	Nanoparticles	Gold Nanorods and liposomes	Tetrodotoxin and Dexmedetomidine:	2016 Jun	[179]
Cancer pain	Nanoparticles	Ethosomal gel	Meloxicam	2014 Jun	[180]
Cancer pain	Nanoparticles	F127 hydrogel	clonidine and ropivacaine	2022 Jun	[141]
Cancer pain	Nanoparticles	Hydrogel	Dexmedetomidine and bupivacaine	2018 Oct	[140]
Cancer pain	Nanoparticles	Thermo-sensitive hydrogel	Levobupivacaine	2023 Aug	[181]
Cancer pain	Extracellular vesicles	Niosomes	Ibuprofen and Lidocaine	2017 Dec	[182]
Cancer pain	Other delivery systems	DNA aptamers	tetrodotoxin and saxitoxin	2023 Apr	[183]
Immunotherapy	Nanoparticles	PLGA	Riboxxim	2021 May	[144]
Immunotherapy	Nanoparticles	a lipid calcium phosphate nanoparticle	pDNA of an engineered PD-L1 trap and/or CXCL12 trap	2017 Oct	[146]
Immunotherapy	Nanoparticles	Fe3O4-siPD-L1@M-BV2	siPD-L1	2022 Mar	[184]
Immunotherapy	Nanoparticles	pH-responsive nanoliposome	Doxorubicin, R848 and losartan	2023 May	[185]
Immunotherapy	Nanoparticles	Ferumoxytol nanoparticles	Monophosphoryl lipid A	2023 Apr	[186]
Immunotherapy	Nanoparticles	Anti-CD11b- and IR820-conjugated bovine serum albumin nanoparticles	Decitabine	2023 May	[187]
Immunotherapy	Nanoparticles	Nanoparticles	CTLA-4 small interfering RNA (siRNA)	2016 Jun	[148]
Immunotherapy	Nanoparticles	liposomes	Paclitaxel derivative and BMS-202	2022 Jan	[188]
Immunotherapy	Nanoparticles	polymeric micelles	IDO inhibitor	2023 Nov	[189]
Immunotherapy	Nanoparticles	lipid-polymer hybrid drug delivery system	JQ1, gemcitabine elaidate, and pIL-12	2023 Jul	[190]
Immunotherapy	Nanoparticles	nanoformulation	Nano-Folox and Nano-FdUMP	2021 Jan	[149]
Immunotherapy	Nanoparticles	Papaya Mosaic Virus-Derived Nanoparticles	/	2016 Mar	[151]
Immunotherapy	Nanoparticles	high-density lipoprotein-mimicking nanodiscs	Antigen peptides and adjuvants	2017 Apr	[152]
Immunotherapy	Nanoparticles	Mesoporous polydopamine	Metformin and toll-like receptor 7/8 agonist resiquimod	2024 Mar	[154]
Immunotherapy	Nanoparticles	magnetic mesoporous silica nanoparticles	doxorubicin	2018 Nov	[155]
Immunotherapy	Nanoparticles	Poly (lactic-co-glycolic acid) nanoparticles	Resiquimod(R-848)	2022 Dec	[157]
Immunotherapy	Nanoparticles	ZnS@BSA Nanoclusters (Bovine Serum Albumin)	Not specified	2021 Dec	[191]
Immunotherapy	Nanoparticles	red blood cells (RBCs)-based gel	Imiquimod (R837) adjuvant	2021 Apr	[192]
Immunotherapy	Nanoparticles	nanovehicles	SR-717and a STING agonist	2023 Aug	[193]
Immunotherapy	Microneedle	Microneedle	Anti-PD1 antibody and 1-methyl-dl-tryptophan	2016 Sep	[147]
Immunotherapy	Microneedle	Polymeric hollow-structured microneedle patch	Anti-programmed death-ligand 1 antibody	2020 Feb	[194]
Immunotherapy	Microneedle	Self-locking microneedle patches	SD-208 and anti-PD-L1 antibody	2023 Mar	[195]
Immunotherapy	Live cells	liquid nitrogen-treated AML cells	doxorubicin	2020 Dec	[196]
Immunotherapy	Blood platelets	Platelets engineered to deliver anti-PD-L1 antibodies	Anti-PD-L1 checkpoint antibody	2019 Jun	[197]
Immunotherapy	Extracellular vesicles	Cellular membrane nanovesicles platform	PD-1/PD-L1 inhibitor BMS	2022 Mar	[198]
Immunotherapy	Extracellular vesicles	Bone marrow mesenchymal stem cell-derived nanovesicles	Photosensitizer and immune adjuvant (R837)	2022 Feb	[199]
Immunotherapy	Extracellular vesicles	Radiation-treated cell-released microparticles	USP7 inhibitor	2023 Mar	[200]
Immunotherapy	Extracellular vesicles	Bacterial outer membrane vesicle	/	2020 Jan	[201]
Immunotherapy	Bacteria	Modified engineered bacteria	NHS-N782 and JQ-1	2022 Jul	[202]

siPD-L1: small interfering RNA against PD-L1; BMS-202: a small molecule PD-1/PD-L1 inhibitor; SR-717: a photothermal agent; SD-208: TGF-β inhibitor; NHS-N782: a heptamethine cyanine dye; JQ-1: a bromodomain and extraterminal domain inhibitor.

## 5 Conclusion

Advancements in tumor research have revealed that modern medicine must address not only the eradication of tumor cells but also the management of tumor-related complications. These complications can cause significant suffering and risks for patients. DDSs provide innovative strategies and techniques for treating tumor-related complications, with an emphasis on enhancing treatment precision and safety. The rapid development of DDSs has introduced new strategies and methods for treating these complications, aimed at enhancing the precision and safety of treatments and ultimately improving patient quality of life and survival rates.

Tumor complications, including cancer-associated thrombosis, malignant pleural effusion, tumor-related infections, and cancer pain, significantly threaten patients’ quality of life and survival. Traditional treatments for these complications, while somewhat effective, are often accompanied by significant side effects and limited therapeutic responses. DDSs enhance the efficacy of traditional therapies by optimizing drug distribution within the body, improving targeting, and controlling the rate of drug release, thereby minimizing the toxic side effects associated with treatment. From nanotechnology to cell-based delivery strategies, various DDSs provide expansive prospects and unlimited potential for innovation in the treatment of tumor complications. In particular, DDSs show immense potential in overcoming drug insolubility, enhancing drug concentrations within the tumor microenvironment, achieving tumor-specific targeting, and reducing systemic side effects. Despite the substantial potential of DDSs in cancer therapy, significant challenges remain, which include effectively delivering drugs to hard-to-reach tumor sites, navigating the complexities of the tumor microenvironment that impact drug delivery, and minimizing potential toxicity and adverse reactions caused by DDSs. Further research is required to optimize drug delivery pathways, enhance delivery efficiency and accuracy, and lower production costs. Additionally, creating personalized drug delivery strategies tailored to various types of tumor complications is crucial for realizing precision medicine and improving therapeutic outcomes.

In conclusion, advancements in drug delivery technology provide significant opportunities in the treatment of tumor complications, offering cancer patients a broader range of therapeutic options and signaling a shift towards more efficient and personalized cancer management. Along with the advance of technology and research through, the integration of the interdisciplinary cooperation, we can expect the future cancer patients will anticipate more precise and more secure treatment options. These advancements will contribute to improving patient quality of life, extending survival, and ushering in a new era of cancer care.

## CRediT authorship contribution statement

**Kerui Li:** Writing – original draft, Project administration, Data curation. **Bei Guo:** Writing – original draft. **Junmou Gu:** Writing – original draft, Funding acquisition. **Na Ta:** Writing – original draft, Conceptualization. **Jia Gu:** Investigation, Conceptualization. **Hao Yu:** Supervision, Project administration. **Mengchi Sun:** Visualization, Supervision. **Tao Han:** Supervision, Project administration, Funding acquisition, Conceptualization.

## Availability of data and materials

Not applicable.

## Ethics approval and consent to participate

Not applicable.

## Funding

10.13039/100014717National Natural Science Foundation of China NO.82473360; Natural science Foundation project of Liaoning Province 2021-MS-183; 10.13039/501100007765Shenyang Science and Technology Bureau Project NO.23-506-3-01-30; Shenyang Science and technology project funding No.22-321-33-06.

## Declaration of competing interest

The authors declare that they have no known competing financial interests or personal relationships that could have appeared to influence the work reported in this paper.

## Data Availability

No data was used for the research described in the article.
